# Bridging the gap: Multi‐stakeholder perspectives of molecular diagnostics in oncology

**DOI:** 10.1002/1878-0261.70103

**Published:** 2025-08-14

**Authors:** Jorine Arnouts, Senada Koljenović, Elise Daems, Karolien De Wael, Marc Peeters, Léon C. van Kempen, Greetje Vanhoutte, Karen Zwaenepoel, Timon Vandamme

**Affiliations:** ^1^ Department of Pathology Antwerp University Hospital Edegem Belgium; ^2^ Multidisciplinary Oncological Center Antwerp (MOCA) Antwerp University Hospital Edegem Belgium; ^3^ Center for Oncological Research (CORE) University of Antwerp Wilrijk Belgium; ^4^ A‐PECS, Department of Bioscience Engineering University of Antwerp Belgium; ^5^ NANOlight Center of Excellence University of Antwerp Belgium

**Keywords:** molecular diagnostics, oncology, stakeholders, unmet clinical needs

## Abstract

Molecular diagnostics has revolutionized cancer management, enabling the identification of diagnostic, prognostic, and predictive biomarkers. Despite advancements in technologies such as whole genome sequencing, their translation into clinical practice remains challenging due to insufficiently demonstrated clinical utility. This study identifies unmet clinical needs and requirements for innovative molecular technologies in oncology through interviews (*n* = 22) and an online survey (*n* = 116), gathering insights from hospital professionals, industry representatives, and health policy and quality assessment experts. Our findings emphasize the increasing importance of liquid biopsies (LBx), particularly plasma‐based assays. Key unmet needs in this area include therapy response monitoring, minimal residual disease detection, and predictive biomarker testing. Additionally, we outline technology requirements tailored to diverse clinical biomarker applications and both centralized and decentralized laboratory settings. A central challenge lies in achieving an optimal balance between multiplexing capacity and turnaround time. By bridging the gap between technology development and real‐world application, this study paves the way for the implementation of new molecular technologies that better meet the needs of the oncology community, ensuring clinical utility and ultimately improving patient care.

## Graphical abstract

Although molecular diagnostics is transforming cancer care, implementing novel technologies remains challenging. This study identifies unmet needs and technology requirements through a two‐step stakeholder involvement. Liquid biopsies for monitoring applications and predictive biomarker testing emerge as key unmet needs. Technology requirements vary by clinical application and laboratory type, with balancing multiplexing and turnaround time (TAT) as a major challenge.

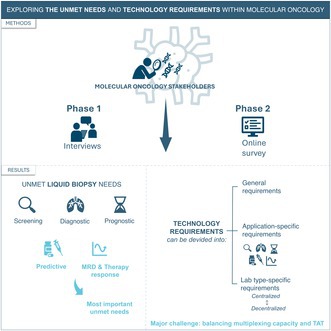

**Abbreviations**
cfDNA: cell‐free DNA
CSF: cerebrospinal fluid
ctDNA: circulating‐tumor DNA
dPCR: droplet PCR
FFPE: formalin‐fixed paraffin‐embedded
LBx: liquid biopsy
MRD: minimal residual disease
NA: nucleic acid
NGS: next‐generation sequencing
P: participant
PCR: polymerase chain reaction
qPCR: quantitative PCR
SD: standard deviation
TAT: turnaround time
WES: whole exome sequencing
WGS: whole genome sequencing

## 1 Introduction

Molecular diagnostics has become a cornerstone in cancer management, enabling the identification of diagnostic, prognostic, and predictive biomarkers to aid in diagnosis, refine prognosis, and guide targeted therapy selection, respectively [1, 2, 3]. Biomarker detection typically involves nucleic acid (NA) analysis using techniques like quantitative PCR (qPCR) and targeted next‐generation sequencing (NGS) on formalin‐fixed paraffin‐embedded (FFPE) tissue samples. This field underwent a major revolution from the 1970s onwards, progressing from Sanger sequencing and PCR to targeted NGS [1, 2, 4, 5]. Building on these advancements, the field continues to evolve, driven by innovations in comprehensive sequencing technologies like comprehensive genomic profiling (CGP) and whole‐genome sequencing (WGS), alongside the development of novel approaches such as single‐cell sequencing, long read (Nanopore®) sequencing, CRISPR‐based diagnostics, and biosensor technologies [2, 6, 7]. An area of growing interest is liquid biopsy (LBx) testing, particularly cell‐free DNA (cfDNA) analysis in bodily fluids such as plasma, urine, and cerebrospinal fluid (CSF) [1, 2, 4, 5, 8]. Since 2016, five plasma‐based LBx assays have received FDA approval as companion diagnostics. In addition, numerous LBx tests are in development, expanding beyond predictive biomarkers to include applications such as minimal residual disease (MRD) detection, therapy response monitoring, and cancer screening [1, 2, 5, 8, 9, 10].

Translating new molecular technologies from bench to bedside remains challenging, primarily due to the lack of demonstrated clinical utility [8, 11, 12, 13, 14]. This is defined as the likelihood of the test results to guide clinical decisions that improve patient outcomes [3]. Multiple studies have emphasized this challenge of clinical utility, for instance in the implementation of WGS, circulating‐tumor DNA (ctDNA) analysis, blood‐based early cancer detection tests, and MRD detection [11, 15, 16, 17]. Addressing clinical utility requires efforts early in the development process of molecular technologies and starts with the identification of unmet clinical needs [3, 8, 18, 19, 20, 21]. This involves a thorough evaluation of current practices to identify areas in need of more efficient or practical diagnostic and management solutions. If a new technology is intended to replace an existing one, it is crucial to demonstrate how it surpasses current technologies in terms of performance, invasiveness, cost‐effectiveness, or turnaround times (TATs) [3, 19, 21].

This study aims to address this challenge by identifying the unmet clinical needs in molecular oncology diagnostics and establishing the requirements for new molecular technologies to ensure their clinical utility. To achieve this, a detailed stakeholder analysis was conducted, involving different hospital professionals, policy makers, quality assurance experts, and industry representatives. A two‐phase process was used, including an interview phase to gather initial insights and an online survey phase to further refine and quantify the identified unmet needs and requirements. By bridging the gap between technology development and clinical adoption, we can pave the way for the introduction of new molecular technologies that better meet the needs of the oncology community and ultimately improve patient care.

## 2 Materials and methods

**Fig. 1:**
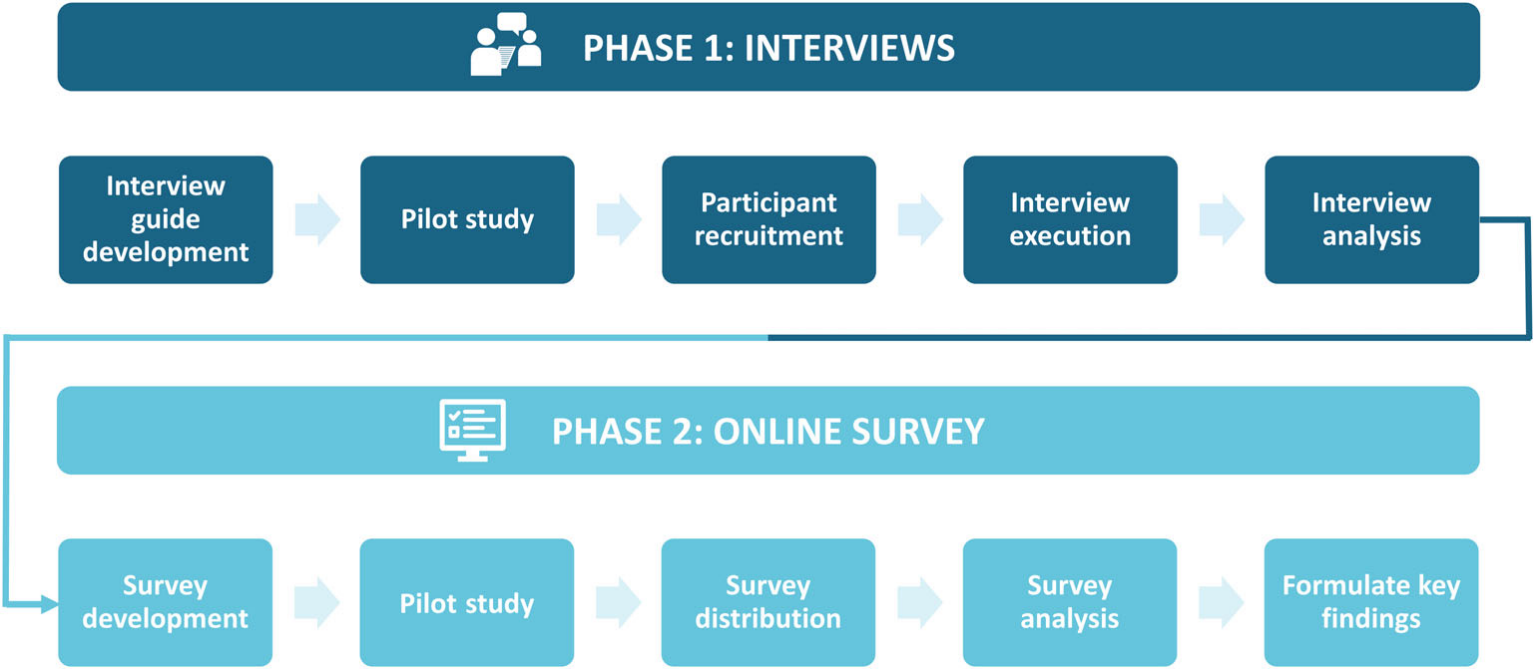
Overview of study design. Two‐phase process: Phase 1 (dark blue) involved developing the interview guide, conducting a pilot with stakeholders, recruiting participants, performing interviews, and analyzing the results. Phase 2 (light blue) included designing an online survey from interview findings, piloting it, distributing the survey, analyzing responses, and summarizing key findings.

This study employed an exploratory sequential mixed methods design. Qualitative insights were first obtained through semi‐structured, in‐depth interviews, and these findings were refined and quantified by conducting an online survey (Fig. 1). Both phases were approved by the independent Ethics Committee for the Social Sciences and Humanities (EASHW) of the University of Antwerp (SHW_2024_122).

### 2.1 Interview phase

An interview guide (Appendix S1) was developed with input from different experienced molecular oncology stakeholders: an oncologist, a pathologist, a clinical biologist, a molecular biologist, and a laboratory technician. A pilot interview with these experts was conducted to refine the guide. The final guide included questions on personal background, current practices in molecular diagnostics (e.g., applications, sample types, technologies), and future needs and innovations. Each participant received a customized set of questions based on their profession.

Purposive sampling was used to recruit international stakeholders from diverse backgrounds (different professions, countries, work settings, experience, and domain focus). Target groups included oncologists, pathologists, molecular biologists, clinical biologists, molecular technicians, as well as professionals from the health policy, quality assessment, and industry sectors. Participants were selected through the research team's network and invited via email. All participants provided informed consent for interview audio recording, transcription, and use of the data. Interviews of 30 to 60 min were conducted between January and March 2024 in either Dutch or English and were held in person or online through the Teams platform. Data collection continued until thematic saturation was reached.

Interviews were transcribed verbatim and personal information was pseudonymized. The transcripts were analyzed in nvivo 14 software [22] using Braun and Clarke's thematic analysis framework (data familiarization, coding, theme identification, and interpretation) [23]. Coding was performed both deductively (based on predefined interview topics) and inductively (emerging from the data). Basic descriptive statistics were applied to the sociodemographic data using ibm spss statistics 29 [24].

### 2.2 Online survey phase

The online survey (Appendix S2), developed using Qualtrics [25] and based on interview findings, was piloted with an oncologist, pathologist, and clinical biologist to ensure clarity and relevance. It comprised three sections: demographics, unmet needs, and requirements for molecular technologies. Demographic questions covered specialty, subspecialty, country, and experience. Unmet need questions focused on the implementation of molecular diagnostic applications, including LBx testing for various biomarker applications. The requirements section addressed preferences for sample types, (de)centralization, TAT, multiplexing, and key technology features across different applications. Responses were collected via multiple‐choice, 5‐point Likert scale, and ranking questions. The networks of the research team members and various international working groups were utilized to distribute the survey via social media platforms, newsletters, emails, and in‐person at conferences to reach a diverse respondent pool. The survey was available from May 27, 2024, to August 31, 2024. Informed consents of participants were obtained at the beginning of the survey. All data were completely anonymous.

Survey analysis was conducted using ibm spss statistics 29 [24]. Descriptive statistics (mean and standard deviation (SD)) were applied to sociodemographic data and responses to multiple‐choice, multiple‐response, and 5‐point Likert scale questions. Likert responses were scored from 1 (strongly disagree) to 5 (strongly agree). For ranking questions, scores of 1, 2, and 3 were given to items ranked first, second, and third, while unranked items received a score of 4. Mean scores were calculated to determine final rankings for both question types. Visualization of the data was performed in Microsoft Excel.

## 3 Results

### 3.1 Interview phase

#### 3.1.1 Interview participants

Twenty‐two molecular oncology stakeholders agreed to participate in the interview phase out of the 32 invited. The participants (P) represented various professional groups, including oncologists (*n* = 5), pathologists (*n* = 5), clinical biologists (*n* = 2), molecular biologists (*n* = 2), molecular laboratory technicians (*n* = 3), industry representatives (*n* = 4), and health policy (*n* = 1) and quality assessment experts (*n* = 1). Table 1 provides an overview of the participants' characteristics. The mean professional experience was 15.45 years (range: 4–25 years).

**Table 1:** Interview participants' characteristics. BE, Belgium; CRO, contract research organization; EQA, external quality assessment; FR, France; H&N, head and neck; IT, Italy; NET, neuroendocrine tumors; NL, the Netherlands; P, participant; UK, United Kingdom.

P	Profession	Experience (years)	Work setting	Country	Domain focus
P1	Oncologist	11	University hospital	BE	Digestive, NET
P2	Oncologist	21	Non‐University & University hospital	BE	Digestive, NET, H&N
P3	Oncologist	10	Non‐University hospital	BE	Urological
P4	Oncologist	25	University hospital	FR	H&N
P5	Oncologist	20	University hospital	IT	H&N, melanocytic
P6	Pathologist	4	University hospital	BE	Neuro
P7	Pathologist	10	University hospital	BE	Thoracic, endocrine
P8	Pathologist	15	University hospital	UK	H&N
P9	Pathologist	20	University hospital	BE	Molecular pathology
P10	Pathologist	17	University hospital	BE	H&N, melanocytic
P11	Clinical biologist	20	University hospital	BE	Molecular pathology
P12	Clinical biologist	25	Non‐University hospital	BE	Molecular pathology
P13	Molecular biologist	25	University hospital	NL	Molecular pathology
P14	Molecular biologist	15	University hospital	BE	Molecular pathology
P15	Laboratory technician	10	University hospital	BE	Molecular pathology
P16	Laboratory technician	7	Industry: CRO	BE	Research
P17	Laboratory technician	23	University hospital	NL	Molecular pathology
P18	Industry expert	13	Industry: CRO	BE	Research
P19	Industry expert	20	Industry: diagnostic	BE	Diagnostics
P20	Industry expert	5	Industry: diagnostic	BE	Diagnostics
P21	Policy expert	11	Health institute	BE	Health policy
P22	Quality assessment expert	13	EQA	UK	Health quality

#### 3.1.2 Current clinical practice of molecular diagnostics in oncology

##### 3.1.2.1 Current applications

Participants highlighted the growing importance of molecular diagnostics in clinical oncology, identifying three key areas of contribution, as summarized by one participant: ‘I would say three areas. The first and most important one of those has been obviously therapy selection to determine which drugs are suitable for a particular patient. The second is aiding in diagnosis to determine the exact disease. And then there is a small part of prognosis in there’ (P22). As part of therapy selection, participants also referred to the use of molecular results to guide inclusion in clinical trials: ‘Does the patient have a target for which we have a reimbursed treatment or not? If it is reimbursed, fine. If not, we often look for studies we are running ourselves or that are being conducted in a nearby center.’ (P2) Furthermore, they noted that its application varies depending on the tumor type: ‘So within the head and neck specifically, I think that it is rather more limited than it is in some other body sites’ (P6).

##### 3.1.2.2 Currently used molecular technologies

PCR‐based and NGS‐based technologies were highlighted as the main techniques for molecular diagnostics. PCR methods include qPCR, droplet PCR (dPCR), and fast PCRs such as Idylla™, while NGS methods include targeted NGS, whole exome sequencing (WES), and WGS, both DNA and RNA‐based. Participants agreed that although PCR was historically dominant, targeted NGS has now taken the lead, primarily due to its multiplexing capabilities. However, they also highlighted challenges associated with NGS, such as high costs, the need for adequate sample material, and the requirement for specialized infrastructure and expertise. Furthermore, the longer TAT of NGS was a concern, with oncologists voicing particularly strong opinions on this matter. ‘If the tissue is processed in‐house, is of sufficient quality, and the test is routine, we can have the results within a week. However, I often have to tell a patient that three weeks have passed, and we still don't have results, which is very frustrating.’ (P1) The need to batch samples, while crucial for reducing costs, contributes to the extended TAT. Its complexity and dependence on batching drive the centralization of NGS technologies, which in turn also extends the TAT. WES and WGS were implemented in only a limited number of centers, where the challenges related to targeted NGS are even more pronounced. PCR technologies, on the other hand, were praised for being relatively fast, cheap, and less complex, though with low multiplexing capabilities and throughput. dPCR, known for its high sensitivity, was specifically mentioned as a valuable tool for LBx analysis. Overall, NGS was considered more efficient, requiring less material and time than running multiple separate PCR tests.

##### 3.1.2.3 Currently used sample types

All participants identified FFPE tissue as the current gold standard for molecular testing. They noted that FFPE samples can be stored long‐term but are of lower NA quality. In contrast, fresh frozen tissue (FFT), used to a lesser extent, offers higher DNA and RNA quality and is preferred for WGS. Participants identified three general drawbacks of tissue samples: they are invasive, often yield insufficient tumor tissue, and may not fully represent the entire tumor. Cytological samples and LBx, particularly cfDNA from plasma, were also mentioned but are used far less frequently: ‘Most tests involve FFPE tissue, including both small biopsies and larger resection specimens. We also receive cytological preparations, either liquid‐based or on slides, and liquid biopsies, which are primarily blood‐based’ (P13). LBx are only used in limited scenarios: ‘In experimental settings or when no tissue is available, we sometimes use liquid biopsies as an alternative, but this is not yet standard practice’ (P1). dPCR and NGS are the primary technologies used for LBx tests. Participants identified two main challenges hindering the wider adoption of LBx testing: ‘The biggest issue is the sensitivity of the tests, particularly for detecting low disease loads, which often fall below the detection threshold. Additionally, the lack of reimbursement acts as a practical barrier and is one of the main reasons these tests are not more widely requested’ (P7).

#### 3.1.3 Emerging applications as unmet needs in molecular oncology

Three applications were predominantly highlighted as unmet clinical needs, summarized by one participant: ‘Perhaps in the field of liquid biopsies for minimal residual disease assessment, therapy response monitoring, and certainly primary cancer screening’ (P12). Therapy response monitoring involves tracking changes in tumor burden over time to assess treatment effectiveness, while MRD focuses on measuring residual cancer cells at very low levels to evaluate relapse risk. For MRD, participants pointed out that current tests are largely tumor‐informed and rely on time‐consuming, costly tissue‐based sequencing. Tumor‐agnostic approaches, particularly those based on methylation, show promise but remain in the early stages of development.

Screening (i.e., detecting cancer at an early stage in asymptomatic individuals), with in particular multi‐cancer screening, was frequently described as the ‘holy grail’ of the field: ‘Imagine being able to say, using this technique, we'll take a blood sample and alongside your cholesterol check we'll see if there are any tumors. There are already some efforts underway, and that would, of course, be fantastic’ (P11). However, not all participants shared this enthusiasm, with one commenting, ‘Screening? We're not going there. We'll never have a pan‐cancer screening tool’ (P1). A pronounced single‐cancer screening need was also highlighted: ‘One important need is pancreatic cancer. It's just a huge medical need, because diagnosis is often made at a late stage, and the disease is just horrible. If we could have a test like the fecal test used for colorectal screening that would be a major advancement’ (P4).

#### 3.1.4 Requirements for novel technologies in molecular oncology

##### 3.1.4.1 Sample type compatibility

Participants agreed that any new molecular technology must be compatible with FFPE tissue: ‘I think we are still living in an FFPE world, so you have no choice but to ensure your test works on FFPE’ (P1). FFT is gaining significance, particularly with the rising interest in WGS. Cytological samples were regarded as beneficial but less essential. LBx were recognized by participants for their key advantages, as they are less invasive, easier to collect, and suitable for repeated testing. Many highlighted their potential, but there was no clear consensus on their future role. The majority of stakeholder groups were enthusiastic about a shift toward LBx for current (e.g., predictive biomarkers) and future applications (e.g., MRD biomarkers) with one oncologist stating, ‘We should move more toward liquid biopsies, as they are easier to collect and capture tumor heterogeneity’. (P2) Pathologists, on the other hand, were more cautious, believing that tissue biopsies will continue to play a critical role: ‘I believe the real value of liquid biopsies lies primarily in patient follow‐up. In the initial workup, or for a large portion of patients, there will still be an advantage in starting with a solid tissue biopsy, especially when technically feasible’ (P7). Plasma was identified as the most relevant bodily fluid for LBx, followed by urine, CSF, and saliva.

##### 3.1.4.2 Turnaround time

When discussing desired TATs, many participants emphasized ‘the faster, the better,’ with a TAT of several days, up to 1 week, being acceptable. The acceptable TAT depended on the application, with prompt results being especially crucial for patients with acute illness or in monitoring scenarios. Participants also noted that the transition to WGS could lead to longer TATs and that therefore fast and targeted testing remains critical for specific cases. However, there is no need for true point‐of‐care testing at the patient's bedside for immediate results.

##### 3.1.4.3 Multiplexing

The interviewees were unanimous in the view that multiplexing is essential: ‘Single‐gene testing is clearly no longer an option’ (P12). Multiplexing was highlighted for its efficiency, as it saves time, money, and patient material compared to testing biomarkers individually. Several participants pointed out that the scope of multiplexing depends on the tumor type and the testing application. For instance, in lung cancer, where numerous molecular targets exist, multiplexing was regarded as indispensable. They also emphasized that along with the discovery of multiple biomarkers, the role of multiplexing will in general become more important. However, opinions varied on how broad the testing should be. One oncologist commented, ‘I do my NGS primarily for two or three actionable alterations, and the rest is a nice bonus that I might use later’ (P1). Others, however, advocated for a more comprehensive approach, stating, ‘If you want predictive biomarkers, the more complete you are, the better it is, because some biomarkers appear at very low frequencies in certain cancers’ (P9).

In line with this, some participants noted that broader panels could facilitate access not only to approved therapies but also to experimental treatments. One oncologist, however, emphasized that to fully benefit from such broad testing, there is a need for intelligent systems that help clinicians interpret results and connect them to relevant therapeutic options, including clinical trials and reimbursement status: ‘I hope we'll have systems combining broad molecular testing and AI that can link molecular findings to available treatments or studies. Right now, it's not always clear what's reimbursed or evidence‐based, and I'm sure I've missed opportunities, even if just a few’ (P2).

##### 3.1.4.4 Throughput, batching, and automation

Participants highlighted that the preferred throughput depends on the test duration and the laboratory size. For longer tests, higher throughput is advantageous, enabling faster results and reduced workload. However, it was also noted that achieving high throughput often necessitates batching, which can increase TATs, and requires centralization to ensure an adequate influx of samples. Larger laboratories generally prefer high‐throughput technologies for their efficiency, while smaller laboratories opt to process samples individually if costs allow.

Participants unanimously supported increased assay automation to reduce errors and lower staffing demands. However, full automation in a laboratory setting is not essential. Transparency in data analysis is crucial, as all participants rejected ‘black box’ systems and prefer the ability to also have access to the raw data alongside automated interpretations.

##### 3.1.4.5 Centralization vs. decentralization

The need for specialized expertise and infrastructure was identified as a main reason for technology centralization, particularly for complex tests and rare indications that may not be cost‐effective for small hospitals. Conversely, decentralization offers several benefits such as reduced TATs, improved communication between laboratories and clinicians, and greater knowledge and expertise in smaller centers: ‘I think there are two advantages: it may be faster if you don't have to send and receive tests, and secondly, there's more in‐house knowledge because I often can't go to our pathologists with questions about molecular testing’ (P3). For successful decentralization, participants emphasized the need for standardization, robustness, and automation, as well as user‐friendly systems that are cost‐effective and demand minimal specialized infrastructure.

##### 3.1.4.6 Performance

Participants' opinions on the minimum required diagnostic sensitivity and specificity of a molecular test varied, depending on its intended application. For predictive and diagnostic tests, some participants favored equal diagnostic sensitivity and specificity, while others indicated that sensitivity should be prioritized, ideally close to 100%. For screening tests, diagnostic specificity was emphasized as more critical, with several participants suggesting it should approach 100%. One oncologist noted, ‘If you think you have a tumor when it turns out you don't, that can be psychologically very challenging’ (P3). For LBx, analytical sensitivity is very important, but this importance varies by application and context: ‘In liquid biopsy, if the patient is too sick to take a tissue sample, sensitivity is less of an issue. But for monitoring, especially in cases like MRD, we need it to be extremely sensitive’ (P20).

##### 3.1.4.7 Device features

Smaller devices were generally preferred due to lab space limitations, but size was not deemed critical, with device benefits outweighing size concerns. Touchscreens were seen as convenient but posed usability challenges: ‘Manufacturers are stepping away from touchscreens because people with gloves can't operate them’ (P20). However, keyboards and mice were criticized for being less hygienic in laboratory settings. Opinions on internet connectivity varied; some emphasized the need for offline operation, while others suggested that internet or intranet access should be an option. As one industry expert mentioned, ‘50% of our clients don't want anything in the cloud. Everything has to be offline’ (P20). This preference was largely driven by concerns about data security. The reliance on USB sticks for data transfer was viewed as inconvenient and prone to errors. Finally, several participants highlighted the need for energy‐efficient devices that minimize waste and avoid harmful chemicals.

##### 3.1.4.8 Key considerations for implementation

A primary factor for successful implementation of new tests is regulatory compliance, which varies across regions. In Europe, for instance, adherence to the *In Vitro* Diagnostic Regulations (IVDR) is critical. An industry expert noted that ‘Customers prefer a test with the IVD stamp because it is easier to implement’ (P20). Additionally, compliance with quality ISO standards was frequently mentioned, with ISO 15189 being vital for medical laboratories, and ISO 13485 for medical device developers. Another key factor is the necessity to demonstrate added value over existing alternatives: ‘You need to evaluate whether the added value of the test translates into better patient outcomes or cost savings’ (P7). Furthermore, aligning test prices with existing reimbursement frameworks is crucial for market acceptance.

#### 3.1.5 Future trends in molecular oncology

Participants highlighted the growing importance of molecular diagnostics in oncology, alongside the transformative role of artificial intelligence, particularly in data interpretation, therapy guidance, and refining digital pathology: ‘I think the share of classical conventional microscopy will decrease in favor of molecular diagnostics and image analysis’ (P7). They anticipate molecular technologies becoming faster, more affordable, and more automated, with a shift toward minimally invasive testing: ‘Non‐invasive and minimally invasive testing, that's where there's going to be a huge explosion’ (P22). Advanced sequencing technologies are also expected to expand: ‘I expect that we are moving toward whole genome applications, which are gradually becoming cheaper and cheaper’ (P12). Additionally, interest is growing in broadening the scope of molecular biomarkers, including mitochondrial DNA, microRNA, and a greater focus on methylation and RNA expression markers, alongside with an expansion into multi‐omics approaches: ‘It will not only remain with DNA and RNA; proteomics will certainly be added, and metabolomics will come in as well’ (P21).

### 3.2 Online survey phase

#### 3.2.1 Survey respondents

A total of 116 professionals completed at least 50% of the online survey and were included in the analysis. The respondents' characteristics are summarized in Table 2.

**Table 2:** Survey respondents' characteristics. FISH, fluorescence in situ hybridization; MLPA, multiplex ligation‐dependent probe amplification; NGS, next‐generation sequencing; qPCR, quantitative polymerase chain reaction; WES, whole exome sequencing; WGS, whole genome sequencing.

Respondents: *n* = 116					
**Main specialty**	***n***	**%**		***n***	**%**
Oncologist	17	14.7	Laboratory technician	8	6.9
Pathologist	27	23.3	Health policy expert	1	0.9
Molecular biologist	36	31.0	Industry expert	4	3.4
Clinical biologist	3	2.6	Others^a^	20	17.2
**Subspecialty** ^**b**^	***n***	**%**		***n***	**%**
Brain	19	12.1	Melanoma	12	12.1
Breast	18	16.4	Ovary	22	10.3
Cholangiocarcinoma	33	15.5	Pancreas	16	19.0
Colorectal	16	28.4	Prostate	8	13.8
Endometrium	20	13.8	Sarcomas	12	6.9
Esophageal and gastric	18	17.2	Thyroid	13	10.3
GIST	21	15.5	Urothelial carcinomas	18	11.2
Haemato	37	18.1	Others	13	15.5
Head and neck	26	31.9	Not applicable	13	11.2
Lung	14	22.4			
**Country**	***n***	**%**		***n***	**%**
Armenia	2	1.7	Italy	3	2.6
Australia	1	0.9	Lithuania	1	0.9
Belgium	73	62.9	The Netherlands	6	5.2
Bulgaria	1	0.9	Norway	2	1.7
Croatia	1	0.9	Portugal	1	0.9
Czech Republic	2	1.7	Saudi Arabia	2	1.7
Denmark	1	0.9	Slovenia	1	0.9
France	1	0.9	Spain	1	0.9
India	1	0.9	Sweden	4	3.4
Ireland	3	2.6	Turkey	3	2.6
Israel	1	0.9	UK and Northern Ireland	5	4.3
**Professional experience**	***n***	**%**		***n***	**%**
≤ 5 years	20	17.2	16–20 years	16	13.8
6–10 years	29	25.0	> 20 years	33	28.4
11–15 years	18	15.5			
**Type of center** ^**c**^	***n***	**%**		***n***	**%**
University hospital	59	50.9	(Private) laboratory not present within a hospital's infrastructure	4	3.4
Non‐University hospital	18	15.5	Others	8	6.9
**Is molecular testing performed at your center?**	***n***	**%**		***n***	**%**
Yes	91	78.4	No	25	21.6
**Available technologies** b, d	***n***	**%**	**Free‐text responses**	***n***	
NGS	83	91.2	Optical genome mapping	2	
qPCR	64	70.3	Methylation profiling	1	
FISH	62	68.1	Sanger sequencing	2	
dPCR	45	49.5	MLPA	1	
Fast PCR	31	33.7	Capillary gel electrophoresis	1	
WGS	29	31.9	Rolling circle amplification	1	
Microarray	29	31.9	Whole transcriptome sequencing	1	
WES	23	25.3			

a Free text responses: oral medicine (*n* = 7), surgical oncologist (*n* = 4), clinical scientist (*n* = 2), gastroenterologist (*n* = 2), medicine student (*n* = 2), clinical geneticist (*n* = 1), study coordinator oncology (*n* = 1), hematologist (*n* = 1).

b Multiple response question.

c Asked to pathologists, oncologists, clinical biologists, molecular biologists and laboratory technicians.

d Asked to participants answering ‘yes’ to ‘Is molecular testing performed at your center?’.

#### 3.2.2 Emerging applications as unmet needs in molecular oncology and their likelihood for clinical implementation

**Fig. 2:**
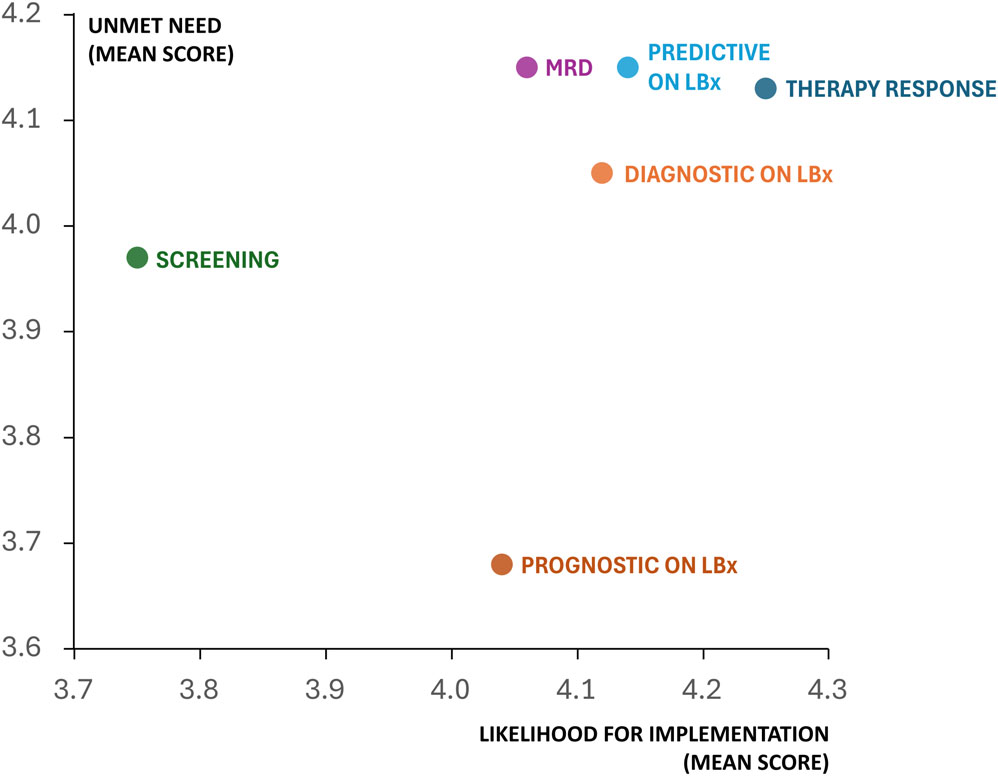
Unmet need vs. likelihood for implementation within the next 5–10 years for different biomarker applications. The *y*‐axis represents the mean Likert scale score indicating the perceived importance of an application as an unmet need. The *x*‐axis represents the mean Likert scale score reflecting the expected likelihood of clinical implementation within 5–10 years. Sample size: *n* = 100–105; missing answers = 11–16. LBx, liquid biopsy; MRD, minimal residual disease.

Based on the mean scores, respondents broadly agreed that testing MRD biomarkers and predictive biomarkers on LBx are the most important unmet needs, closely followed by testing therapy response biomarkers. Other biomarker applications were considered less of an unmet need. Regarding near‐future implementation, respondents were most confident in the adoption of testing therapy response biomarkers within the next 5–10 years, followed by predictive biomarkers on LBx, diagnostic biomarkers on LBx, MRD biomarkers, prognostic biomarkers on LBx, and screening biomarkers, respectively (Fig. 2, Table S1).

#### 3.2.3 Importance of novel molecular technologies' ability to analyze diverse sample types

**Fig. 3:**
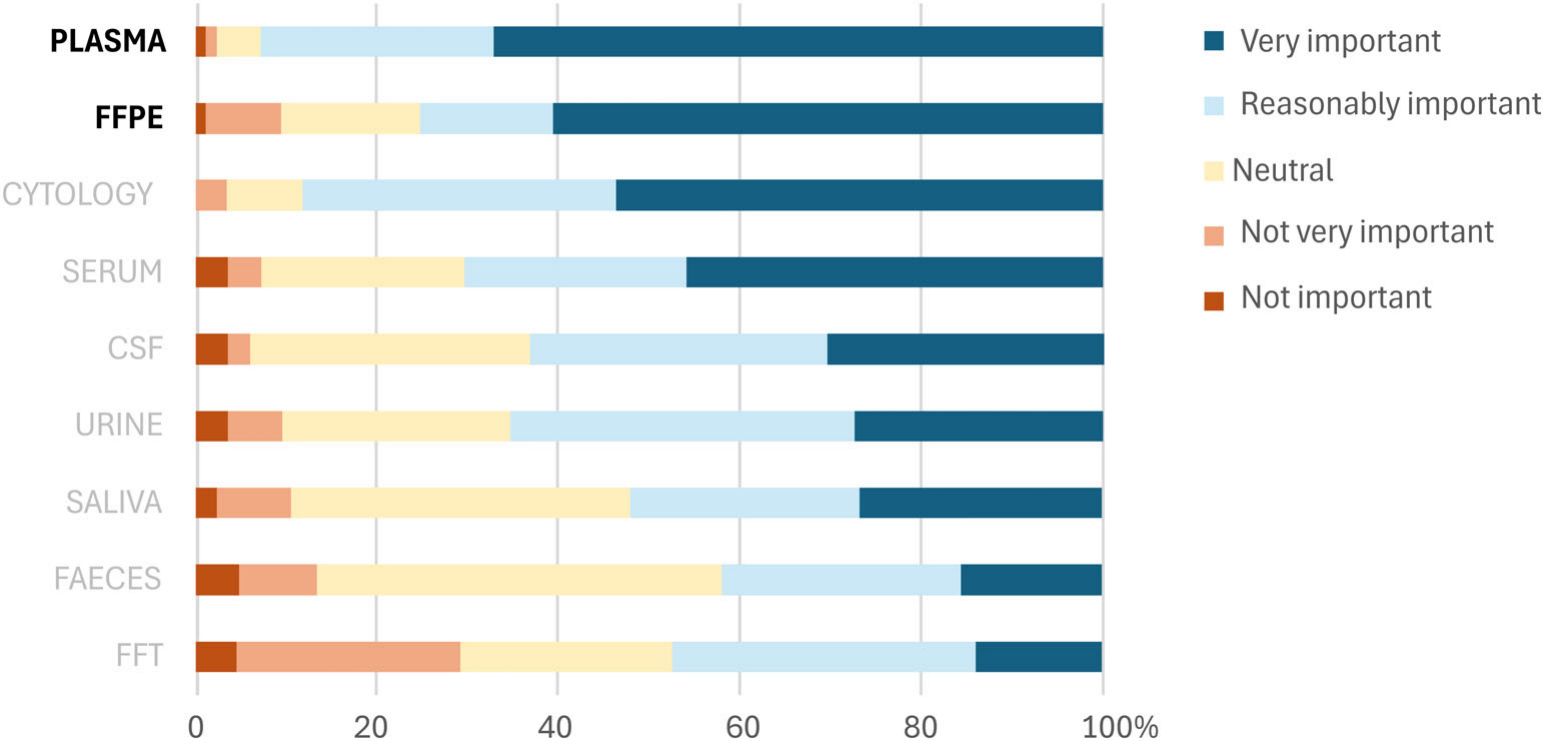
Importance of novel molecular technologies' ability to analyze diverse sample types. The bar charts represent the percentage distribution of responses across five importance levels, indicating how important it is for novel molecular technologies to be compatible with different sample types. Sample size: *n* = 96–99; missing answers = 17–20. CSF, cerebrospinal fluid; FFPE, formalin‐fixed paraffin‐embedded; FFT, fresh frozen tissue.

Plasma was rated as the most important sample type that a novel molecular technology should be able to analyze (58.2% rated it as ‘very important’), followed by FFPE (52.5%), cytology (46.5%), and serum (39.8%). The remaining sample types were generally regarded as less important (Fig. 3).

#### 3.2.4 Importance of molecular technology characteristics across different biomarker applications

**Fig. 4:**
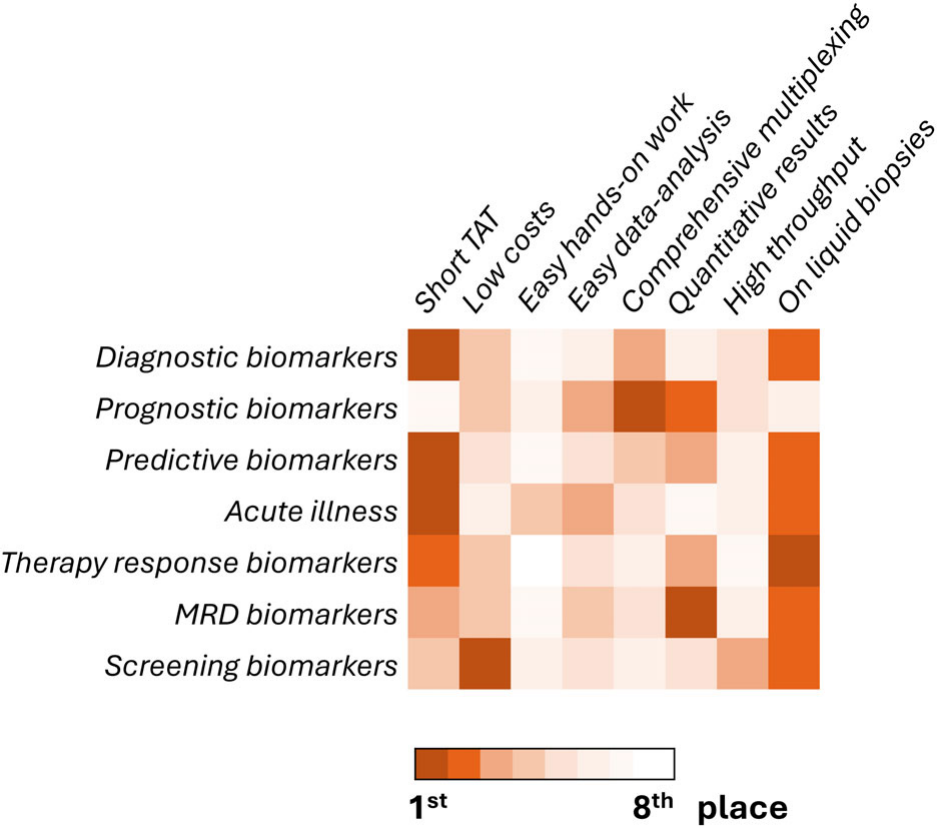
Importance of molecular technology characteristics per biomarker application. Rankings were determined based on the mean scores of each characteristic per application and are visualized in this heatmap. Dark red indicates the highest‐ranked characteristic (1st place), while white represents the lowest‐ranked (8th place). Sample size: *n* = 82–85; missing answers = 31–34. MRD, minimal residual disease; TAT, turnaround time.

The key priorities for diagnostic biomarkers are a short TAT (2.67 ± 1.36), LBx compatibility (3.19 ± 1.11), and comprehensive multiplexing (3.50 ± 0.97), while for prognostic biomarkers these are comprehensive multiplexing (1.72 ± 0.85), quantitative results (1.87 ± 0.76), and easy data analysis (2.03 ± 0.81). For predictive biomarkers the main considerations are a short TAT (3.01 ± 1.30), LBx compatibility (3.28 ± 1.02), and quantitative results (3.41 ± 1.09), but in the context of acute illness, short TAT (2.19 ± 1.36), LBx compatibility (3.32 ± 0.91), and easy data analysis (3.51 ± 0.75) make up the top three. For therapy response biomarkers and MRD biomarkers, the key priorities are LBx compatibility (3.04 ± 1.17 and 3.11 ± 1.16, respectively), short TAT (3.10 ± 1.20; 3.35 ± 1.12), and quantitative results (3.28 ± 1.14; 2.99 ± 1.25), while for screening biomarkers, cost efficiency is the top priority (2.80 ± 1.21), followed by LBx compatibility (3.08 ± 1.11) and high throughput (3.37 ± 1.08) (Fig. 4, Table S2).

#### 3.2.5 TAT vs. multiplexing preferences across different biomarker applications

**Fig. 5:**
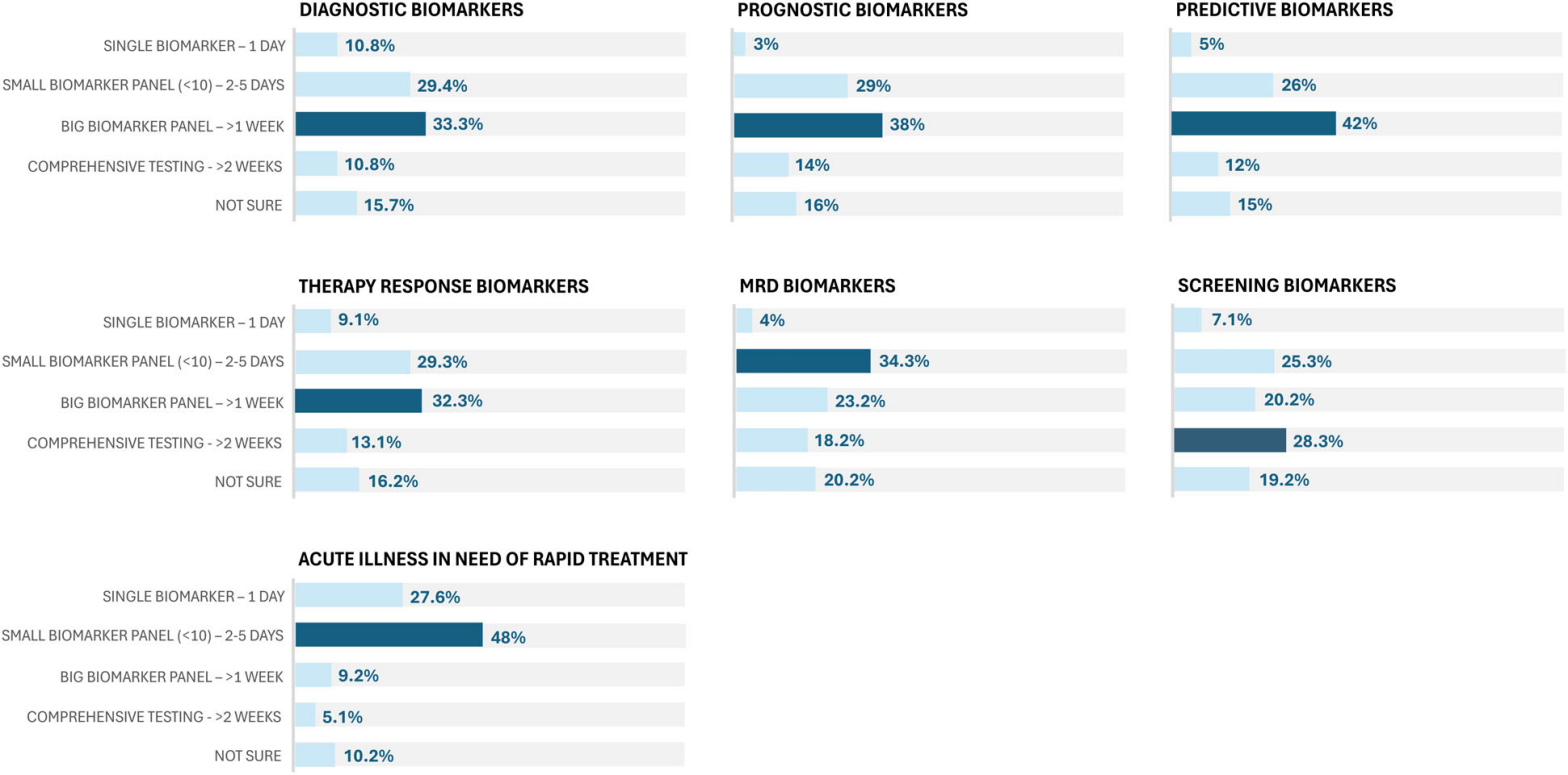
Turnaround time (TAT) and multiplexing preferences across different biomarker applications. The bar charts represent the distribution of respondents' preferences for different TATs and levels of biomarker multiplexing, ranging from single biomarker tests with rapid results to more comprehensive panels with longer turnaround times. A small biomarker panel was defined as one that includes fewer than 10 genes, a large panel refers to assays with multiplexing capacities comparable to targeted next generation sequencing (NGS) (covering 10 to 500 genes), while comprehensive testing encompasses very large panels (> 500 genes) or whole‐genome sequencing (WGS), for instance. Preferences are displayed for different biomarker types, with dark blue indicating the most preferred option. Sample size: *n* = 98–102; missing answers = 14–18. MRD, minimal residual disease.

For diagnostic, prognostic, predictive, and therapy response biomarkers, the majority of respondents preferred large biomarker panels (covering 10–500 genes, e.g., targeted NGS) with a TAT exceeding 1 week, followed by small panels (covering < 10 genes) with a TAT of 2–5 days. For MRD biomarkers, small panels with a TAT of 2–5 days were most preferred, with large panels exceeding 1 week as the second choice. For screening biomarkers, preferences were more evenly distributed, with comprehensive testing (e.g., a panel of > 500 genes or WGS) as the most common choice, closely followed by small panels and large panels. For acute illnesses, nearly half of respondents favored small panels with a TAT of 2–5 days, while about a quarter preferred single biomarker testing within 1 day (Fig. 5).

#### 3.2.6 Centralization vs. decentralization of novel molecular technologies

**Fig. 6:**
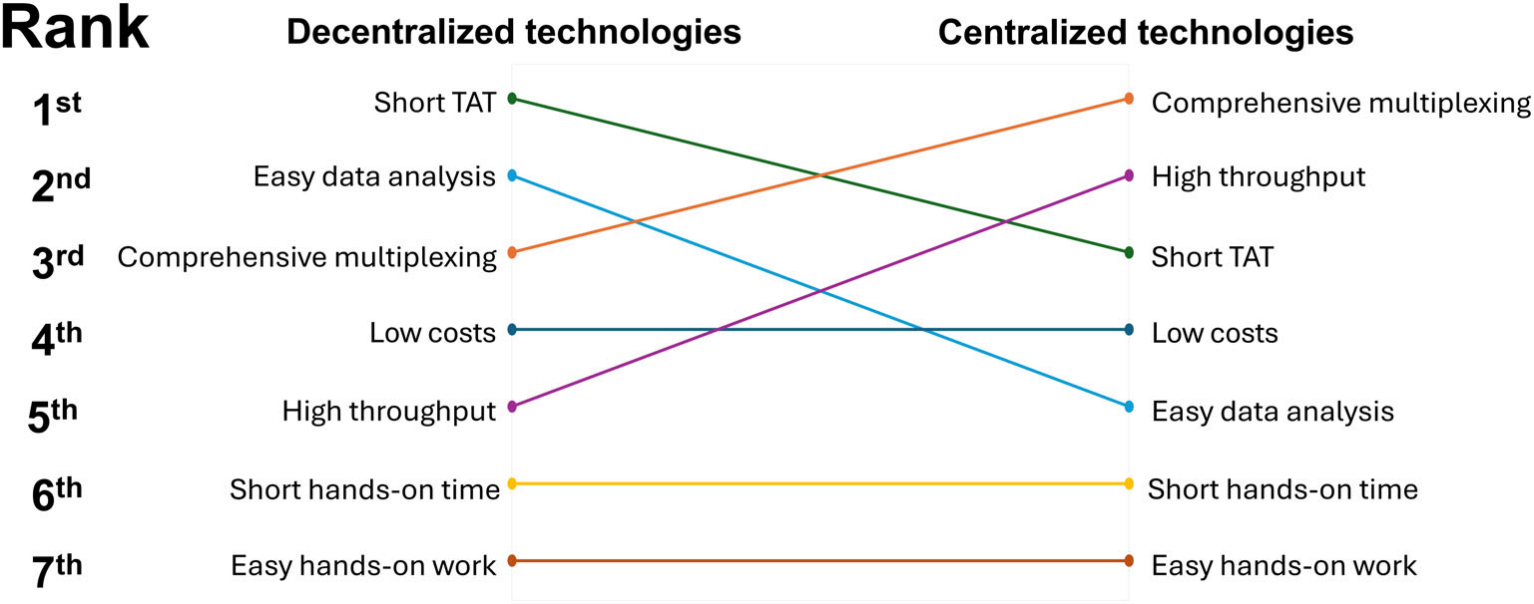
Preferred characteristics for technologies in decentralized and centralized settings. The rankings for each setting are based on the mean scores of different characteristics, with the highest‐ranked characteristic appearing at the top and the lowest‐ranked at the bottom. The figure illustrates how preferences shift between decentralized and centralized settings. Sample size: *n* = 39; missing answers = 0. TAT, turnaround time.

54% of respondents agreed that all novel molecular technologies should be centralized, while 39% disagreed and 7% were unsure. According to the respondents who disagreed with centralization for all technologies, the most important characteristic for technologies in decentralized settings was a short TAT (mean score: 1.90 ± 1.25), followed by easy data analysis (3.03 ± 0.96) and comprehensive multiplexing (3.26 ± 1.09). The top three for technologies in centralized settings were as follows: comprehensive multiplexing was considered most important (2.44 ± 1.23), followed by high throughput (2.46 ± 1.12) and short TAT (2.74 ± 1.25) (Fig. 6, Table S3).

## 4 Discussion

This study connects technological advancements in molecular diagnostics in oncology with the unmet needs in clinical practice by collecting and quantifying information from stakeholders through interviews and an online survey. By interpreting our findings in the context of current practices and emerging trends, we highlight key insights to guide the development and adoption of future molecular diagnostics in cancer care.

In clinical practice, molecular technologies are used to detect diagnostic, prognostic, and predictive biomarkers, with NGS preferred for its efficiency and multiplexing, while PCR remains valued for its speed, cost, and simplicity. The 2024 ESMO guidelines reinforce NGS's importance, recommending it for multiple cancers at advanced or metastatic stages including breast, colorectal, and prostate cancer [26]. Nevertheless, several studies have highlighted inconsistent NGS adoption across countries [14, 27]. From the interviews in this study, multiple barriers to its broader adoption were identified: high costs, the need for specialized expertise and infrastructure, and long TATs. This is in accordance with Horgan et al., identifying expertise and infrastructure deficits as barriers to NGS adoption [28], and Bayle et al. [14] reporting that costs and long TATs often hinder patient access to NGS. Interview participants also foresee a shift toward comprehensive sequencing technologies, which face similar but more pronounced barriers compared to targeted NGS. Indeed, van de Ven et al. [11] emphasized reducing WGS TATs to enable its clinical integration. Of note, the clinical significance of TAT for testing predictive biomarkers depends on when testing is performed during the patient treatment pathway. For testing at the initial diagnosis of advanced disease, TAT may be less critical if results are intended to guide second‐line or subsequent therapies. Conversely, TAT is more critical when testing occurs only after disease progression, as rapid results directly influence timely treatment decisions [29]. Given that guidelines vary by cancer type and region, detailed discussion of local policies falls outside the scope of this study.

Our study has shown that, in addition to current biomarker applications, new applications are on the horizon to address unmet needs. Survey results identified MRD biomarker testing as the most critical unmet need, though it is less likely for short‐term implementation. In contrast, therapy response and predictive biomarker testing on LBx are also critical but highly feasible for near‐term implementation. Screening biomarkers ranked lower with high variation in respondents' opinions (Fig. 2). Similarly, IJzerman et al. and Kramer et al. highlighted MRD and therapy response monitoring as key LBx applications and screening as promising but underdeveloped [15, 30]. The high scores of these LBx applications as unmet needs emphasize the growing importance of LBx testing. Our study has shown that plasma‐based LBx has become the preferred sample type a novel technology should be compatible with, even surpassing FFPE, yet maintaining compatibility with this gold standard remains essential (Fig. 3). In current clinical practice, LBx testing is restricted to cases where tissue samples are unavailable or unsuitable. Interviews identified key barriers to broader adoption, including low sensitivity and reimbursement challenges, aligning with findings from IJzerman et al. [30] and Pascual et al. [17]. Nevertheless, the future role of LBx remains a topic of debate.

**Fig. 7:**
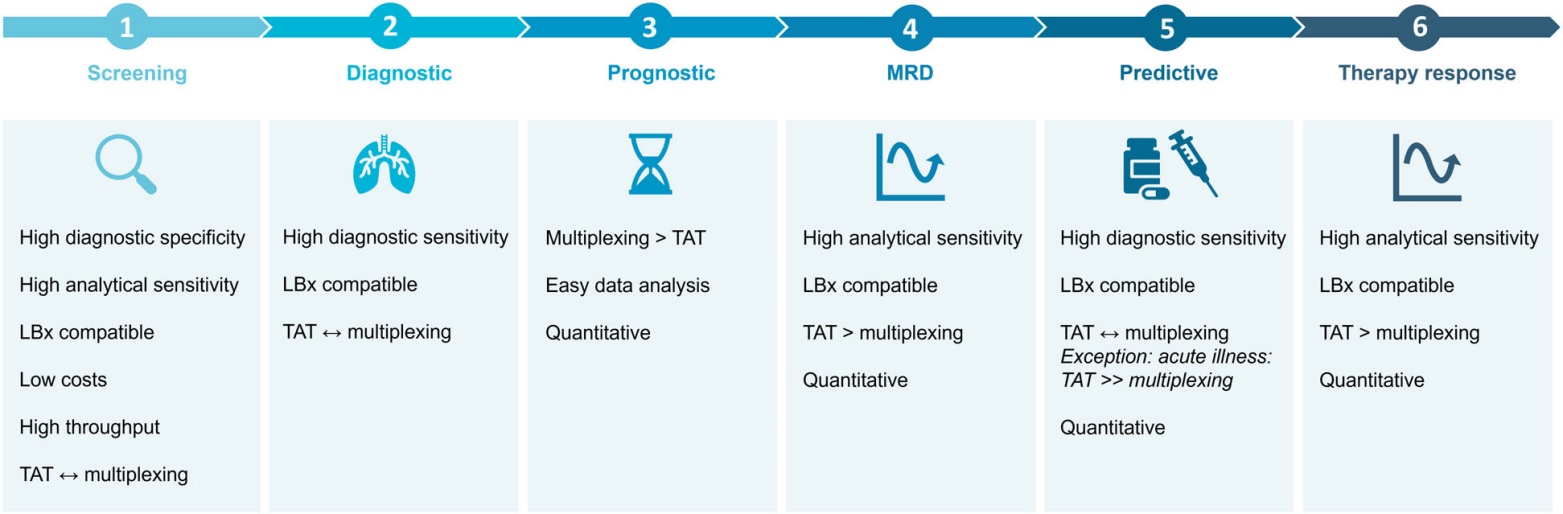
Biomarker application‐specific molecular technology requirements. The symbols indicate the relative preference between sensitivity and specificity. ‘↔’ denotes no clear preference; ‘>’ indicates a moderate preference for either sensitivity or specificity; and ‘≫’ represents a strong preference. LBx, liquid biopsy; MRD, minimal residual disease; TAT, turnaround time.

During the development of novel molecular technologies, it is essential for developers to define their technology's specific applications early on, as our study reveals significant variability in technology requirements between these applications (Fig. 7). For diagnostic and predictive biomarker detection, diagnostic sensitivity and LBx compatibility should be prioritized. Balancing speed and multiplexing is crucial, as both are considered important. Additionally, quantitative results matter for predictive biomarkers. The vast majority of molecular diagnostic tests are designed for FFPE samples, with only five FDA‐approved LBx assays [31], highlighting the unmet need for LBx compatibility. Current LBx assays rely on NGS, with long TATs, or dPCR, with limited multiplexing capacity. Thus, faster technologies with broad multiplexing capabilities are needed. During interviews, acute illness was frequently mentioned as an exception requiring rapid testing and treatment. Nearly half of the respondents preferred smaller panels with a TAT of several days, making dPCR or other rapid, targeted, LBx‐compatible assays well‐suited for such cases. For prognostic biomarkers, covering the broad range of relevant markers is prioritized, while speed is considered less critical since these biomarkers rarely impact immediate patient management. Lastly, quantitative results were also ranked in the top three. Given this, comprehensive sequencing technologies are suitable for detecting prognostic biomarkers.

For MRD and therapy response biomarkers, LBx compatibility is crucial for repeated sampling, while high analytical sensitivity is needed to detect ultra‐low ctDNA levels [17]. Short TATs were prioritized over comprehensive multiplexing, and quantitative results are essential for detecting residual cancer cells and assessing treatment efficacy. Tumor‐informed MRD approaches near clinical practice, like Signatera™, are costly and time‐consuming, combining comprehensive tissue‐based sequencing with personalized LBx testing. Tumor‐agnostic methods, on the other hand, may use predefined broad mutation panels for LBx testing, but growing interest surrounds epigenetic markers like DNA methylation, with both large‐panel sequencing and simpler PCR‐based tests (e.g., Colvera™) in development or already available. These approaches are cost‐effective and offer shorter TATs, better aligning with our MRD technology requirements, enhancing their potential for widespread clinical adoption [32, 33]. Therapy response monitoring using dPCR or NGS is investigated in numerous studies for chemotherapy, targeted therapies, and immunotherapies, but it requires further validation for clinical implementation [17, 34]. Given the importance of short TATs, efforts should focus on accelerating NGS TATs for this application.

For screening biomarkers, high diagnostic specificity is prioritized to limit false positives, as it may result in unnecessary invasive procedures and psychological impact [30]. This impact was already demonstrated by Nadauld et al. [35], who reported transient anxiety increases following false positive results in multi‐cancer early detection tests. On the other hand, high analytical sensitivity is required due to the low ctDNA levels in early disease stages, as well as in tumor types that shed less ctDNA, to limit false negative results [35]. Survey results emphasized the importance of low costs, LBx compatibility, and high throughput. It was the only application where comprehensive testing with a TAT over 2 weeks was accepted, but again with the most variation in responses. Multi‐cancer screening tools, such as the GRAIL Galleri test utilizing comprehensive methylation bisulfite sequencing, show great promise but remain under development. Their high costs, long TATs, and complex analysis currently limit their suitability for population‐based screening. Additionally, it has been shown that the lack of clinical validation in prospective multicenter studies remains a major barrier to their clinical adoption [36]. Therefore, there is an urgent need for the development of more targeted, efficient, and cost‐effective screening assays. Cost importance was also shown by Schroll et al. [16], citing high out‐of‐pocket expenses and lack of reimbursement as key barriers to implementing blood‐based screening assays.

Furthermore, molecular technology developers must consider whether they aim to design for, or whether their technology is best suited to centralized or decentralized settings. Decentralized testing provides faster TATs, better laboratory‐clinician communication, and access to local expertise, but demands automated, user‐friendly, cost‐effective, and fast technologies, while still considering the importance of multiplexing. In contrast, certain tests are more suitable for centralized settings, especially those that are complex, require specialized expertise and infrastructure, involve rare indications, or require batching, but it leads to longer TATs, aligning with Kramer et al. [15]. When developing tests for centralized settings, developers should prioritize high throughput and multiplexing, while still considering the impact of the TAT.

Finally, regardless of the application or laboratory type, key requirements for all near‐future technologies include automation, raw data access, and data security. Furthermore, regulatory compliance, demonstrated added value, and alignment with reimbursement policies are important for final market acceptance. Reimbursement increasingly determines access to molecular technologies. A recent ESMO study by Bayle et al. [14] identified reimbursement as the primary barrier to access across Europe, especially for advanced or high‐cost tests, and revealed significant cross‐country variability, including a lack of a designated authority for test reimbursement in nearly one‐third of countries. Given the heterogeneity of reimbursement systems and our study's primary focus on technology requirements rather than implementation barriers, this aspect lies beyond the scope of the manuscript.

In addition to these practical considerations, another essential aspect of molecular diagnostics is the integration of results into clinical decision‐making, typically facilitated through multidisciplinary discussions, preferably within molecular tumor boards (MTBs) [37]. While our study did not specifically examine that part of the clinical pathway, the inclusion of oncologists, pathologists, clinical and molecular biologists allowed us to capture multiple layers of the decision‐making structure. This multidisciplinary composition is a major strength of the study, as it brings together the opinions of key MTB stakeholders along with laboratory technicians, quality experts, policy makers, and industry representatives, offering broader insights compared to the majority of studies focusing only on clinicians. The integration of qualitative (interviews) and quantitative (survey) data, along with the study's international scope, enhanced its relevance across different contexts and provided a comprehensive overview.

However, several limitations should be considered when interpreting the findings. The relatively small sample sizes restricted statistical analysis. Interview participants were primarily recruited through the research team's professional networks to ensure the inclusion of participants with different backgrounds. However, this may have introduced selection bias. For the survey, partial responses from some participants could have led to response bias. However, missing data were limited as 85% of respondents completed over 80% of questions, and the missing data were evenly distributed across stakeholder groups. Additionally, the uneven distribution of cancer types and professions, with limited industry and health policy representation, a predominance of participants from university hospitals compared to non‐university settings, and the predominantly West‐European participant pool may affect generalizability. Another limitation is the absence of patient involvement, which might have provided insights into access to testing. However, as the study focused on technology requirements and patient knowledge of molecular technologies is generally limited, their inclusion was not deemed appropriate in this context. Additionally, representatives from health technology assessment bodies, regulatory authorities, and payers were asked to complete the questionnaire but were not included in the interview panels. Their limited participation in the questionnaires precludes us from integrating their perspective in this paper, which is a limitation as this could have provided additional insights into reimbursement and policy‐related barriers to test access.

Future research could address these limitations by conducting larger studies with sufficient statistical power to compare different groups. Furthermore, follow‐up research could build on our two‐phase process to explore region‐specific or cancer type‐specific insights, as some factors may vary depending on the cancer type and geographic context.

## 5 Conclusion

In conclusion, this study bridges the gap between technological innovation and real‐world clinical application by providing valuable insights into the unmet needs in molecular oncology and requirements for novel molecular technologies in the field. In doing so, it serves as a strategic guide for technology developers, increasing the chances of successful clinical implementation. While FFPE will remain central to clinical practice in the near term, the use of LBx, particularly plasma, is expected to grow significantly. This growth is driven by the unique advantages of LBx and their broad potential, especially in monitoring applications such as therapy response and MRD, as well as predictive biomarker testing. In addition to some general requirements, molecular technology requirements vary by clinical application and lab type, making it essential to understand these nuances for successful implementation. While multiplexing is essential, the key challenge lies in balancing multiplexing capacity and TAT. As long as this balance is necessary, it will remain essential to differentiate the desired technology requirements based on its context.

## Conflict of interest

TV reports consultancy, advisory roles, honoraria from AstraZeneca, Bayer, Bristol Myers Squibb, Eisai, Elmedix, Ipsen, MyNeoTx, SERB, Novartis, Roche, Sirtex, Servier. Research funding (institutional) from Ipsen and Novartis; support for travel/accommodation from Ipsen, AstraZeneca, Roche, and Servier. The other authors declare no conflict of interest.

## Author contributions

JA was involved in the conceptualization, methodology, formal analysis, investigation, data curation, writing the main manuscript, visualization, interpretation of the data, and reviewing and editing the manuscript. GV, KZ, and TV were involved in conceptualization, methodology, reviewing and editing the manuscript, supervision, and approval of the final version. SK was involved in conceptualization, methodology, reviewing and editing the manuscript, supervision, funding acquisition, and approval of the final version. KDW was involved in project administration, funding acquisition, reviewing and editing the manuscript, and approval of the final version. ED was involved in project administration, reviewing and editing the manuscript, and approval of the final version. LCK was involved in methodology, reviewing and editing the manuscript, and approval of the final version. MP was involved in funding acquisition, reviewing and editing the manuscript, and approval of the final version.

## Supporting information


**Appendix S1.** Interview guide.

**Appendix S2.** Online survey.

**Table S1.** Survey results on emerging applications as unmet needs in molecular oncology and their likelihood for clinical implementation.

**Table S2.** Preferred characteristics for technologies in decentralized and centralized settings.

**Table S3.** Preferred characteristics for technologies across different applications.

## Data Availability

The full interview guide and survey are provided as Appendices S1 and S2, respectively. The interview and online survey data that support the findings of this study are available from the corresponding author (timon.vandamme@uantwerpen.be) upon reasonable request.
