# Supplementary material for: Bridging the gap: Multi‐stakeholder perspectives of molecular diagnostics in oncology
Source: Mol Oncol. 2025 Aug 14;20(2):464–79. doi: 10.1002/1878-0261.70103 (PMC12936412; doi:10.1002/1878-0261.70103)
Supplement: Supplementary file 1 — Appendix S1. Interview guide. [file MOL2-20-464-s004.pdf]

INTERVIEW QUESTIONS

Oncologists

1. General information

- Which organization and department/team do you work for?
- Could you briefly describe your position, your specialism and your main tasks?
- How many years of experience do you currently have? (including and excluding internships during the specialization, if applicable)

2. Current situation

- What does the typical trajectory of your patients look like?
- What is the current role of molecular diagnostics in clinical oncology?
    - Do you request molecular diagnostic tests in your clinical practice?
        - If so, for which tumor types and which biomarkers?
            - For which application(s) do you use the molecular test result? (diagnosis, treatment plan, follow-up...)
            - How often do you request these molecular tests?
            - What molecular diagnostic techniques are used for these markers?
                - What do you think are their pros/cons?
- Which sample types and subtypes do you request molecular testing for? (e.g. tissue: FFPE, FFT, etc.; blood: PMBCs, plasma, serum, ctDNA, CTC, etc.)
    - If liquid biopsies are used:
        - For which application(s) are you requesting molecular testing on a liquid biopsy (primary diagnosis, monitoring of therapy response, minimal residual disease testing, analysis of therapy resistance)?
        - How often do you request such a test?
        - In your opinion, are these tests clinically validated (level 1) or are they still in a clinical research phase?
- What do you think is the current average turnaround time (from taking a biopsy to getting a report) for the biomarkers you request?
    - Are you happy with this?
    - What is ideal and achievable as a TAT?
- Do you believe that molecular diagnostic tests should be centralized in a network?
    - How is this for the currently used tests?
    - What do you think could be an advantage of partially or fully decentralized testing?

3. Demand

- In your opinion, within your specialty, where do you currently see the most need for a new molecular test?
    - For which application(s) and on which (type(s) of) biomarker(s)?
    - What are the requirements for the technology to meet this need?
        - E.g. in relation to speed, cost, sensitivity/specificity, sample type, qualitative/semi-quantitative/absolute quantitative
- Do you think there is a need for a point-of-care test for complex molecular diagnostics? Why (not)?
    - What are pros and cons?
    - What are the requirements for the technique here?

- Which sample types should the test definitely work on and on which sample would it be a bonus? Why?
  - What are the requirements for the test to meet this?
  - If liquid biopsies mentioned:
    - What type of liquid biopsy (saliva, blood, urine, csf)?
    - For which application would you like to request molecular tests on a liquid biopsy?
    - What advantages/disadvantages can liquid biopsies have?
    - In which areas is it possible for the liquid biopsies to (partially) replace the tissue biopsies? Where do tissue biopsies remain a must?
- Do you think it is important for a molecular technique to be able to test multiple biomarkers at the same time? Why?
- What do you think is more important within the various applications: sensitivity (TP/TP+FN) (absence of false negatives) or specificity (TN/TN+FP) (absence of false positives)?
  - What is the minimum diagnostic sensitivity and specificity (in general or per application)?
- How do you see the current clinical practice in oncology in general and in the field of molecular diagnostics evolving? What will it be like in, say, 10 years?
- Is there anything else you would like to add?

Anatomical pathologists/clinical biologists/molecular biologists

1. General information

- Which organization and department/team do you work for?
- Could you briefly describe your position, any specialism and your main tasks?
- How many years of experience do you currently have (including and excluding internships during the pathologist/clinical biologist specialization)? What did you graduate in? (molecular biologists)

2. Current situation

- What is the current role of molecular diagnostics in clinical oncology?
- What molecular diagnostic techniques are used in your laboratory?
    - What are the advantages and disadvantages of these techniques?
    - Which molecular tests are relevant within your specialism?
        - On which tumor types and which biomarkers?
    - For which application(s) is the molecular test result used? (diagnosis, treatment plan, follow-up...)
- What does the standard trajectory look like for a sample that ends up in your laboratory?
- On which sample types and subtypes are molecular tests performed? (e.g. tissue: FFPE, FFT, etc.; blood: PMBCs, plasma, serum, ctDNA, CTC, etc.)
    - If liquid biopsies are used:
        - For which application(s) is molecular testing done on a liquid biopsy (primary diagnosis, monitoring of therapy response, minimal residual disease testing, analysis of therapy resistance)?
        - How often is this done?
        - In your opinion, are these tests clinically validated (level 1) or are they still in a clinical research phase?
- What is your current average turnaround time of complex molecular tests performed in your laboratory?
    - Are you satisfied with that?
    - What is ideal and achievable as a TAT?
- Do you believe that molecular diagnostic tests should be centralized in a network?
    - How is this for the currently used tests?
    - What do you think could be an advantage of partial or fully decentralized testing?
    - What are the requirements for a test that it could still be decentralized?

3. Demand

- In your opinion, within your specialty, where do you currently see the most need for a new molecular test?
    - For which application(s)?
    - On which (type(s) of) biomarker(s)?
    - What are the requirements for the technology to meet this need?
        - E.g. in relation to speed, cost, sensitivity/specificity, sample type, qualitative/semi-quantitative/absolute quantitative
- Do you think there is a need for a point-of-care test for complex molecular diagnostics? Why (not)?
    - What are pros and cons?
    - What are the requirements for the technique here?

- Which sample types should the test definitely work on and on which sample types would it be a bonus? Why?
    - What are the requirements for the test to meet this?
    - If liquid biopsies mentioned:
        - What type of liquid biopsy (saliva, blood, urine, csf)?
        - For which application(s)?
        - What advantages/disadvantages can liquid biopsies have?
        - In what areas is it possible for liquid biopsies to (partially) replace tissue biopsies? Where do tissue biopsies remain a must?
- Do you think it is important for a molecular technique to be able to test multiple samples at the same time? Why?
    - How is this for the current tests?
- Do you think it is important for a molecular technique to be able to test multiple biomarkers at the same time? Why?
    - How is this for the current tests?
- To what extent should a new test be automated? Does it need to be fully/semi-automated?
    - How is this for the current tests?
- What is desirable; the machine that does the interpretation of the data, machine interpretation with supervision of the analysis (requires explainability of the machine), or obtaining raw data and interpreting it yourself?
    - How is this for the current tests?
- Which device size is ideal/acceptable? Do you prefer a portable device or tabletop model?
- What do you think is more important within the various applications: sensitivity (TP/TP+FN) (absence of false negatives) or specificity (TN/TN+FP) (absence of false positives)?
    - What is the minimum diagnostic sensitivity and specificity (in general or per application)?
- How do you see the current clinical practice in oncology in general and in the field of molecular diagnostics evolving? What will it be like in, say, 10 years?
- Is there anything else you would like to add?

Molecular laboratory technicians

1. General information

- Which organization and department/team do you work for?
- Could you briefly describe your position, your specialism and your main tasks?
- How many years of experience do you currently have?

2. Current situation

- What is the current role of molecular diagnostics in clinical oncology?
- What does the standard trajectory look like for a sample that ends up in your laboratory?
- What molecular diagnostic techniques are used in your laboratory?
  - What are the advantages and disadvantages of these techniques?
  - How often are these tests performed in your laboratory?
  - How often do you perform these tests yourself?
  - Which tests are the easiest/most pleasant to perform? Why?
  - What is the average hands-on time of these tests?
    - Are you satisfied with that?
    - What is acceptable as hands-on time?
    - What is ideal and achievable as hands-on time?
  - To what extent are these tests automated?
  - What is the current sample preparation and how long does it take?
  - How many samples are (or can be) tested at the same time with these techniques?
  - How many biomarkers are (or can be) tested at the same time with these techniques?
  - Do you also do data interpretation yourself? How do the devices give a result? Are these already fully interpreted results, e.g. biomarker present/absent or are you receiving raw data or a combination?
- Are mistakes sometimes made during the execution of complex molecular tests, for example the exchange of samples?
  - What kind of errors are these?
  - How do you deal with the risks of making mistakes, i.e. how do you prevent this?
  - Which tests do this happen most frequently?
  - What could be due to this?
  - How could this be prevented in a new technique?
- On which sample types and subtypes are molecular tests performed? (E.g. tissue: FFPE, FFT...; blood: PMBCs, plasma, serum, ctDNA, CTC...)
  - What are the differences in performing tests on the different types of sample? What is the most pleasant?

3. Demand

- Which sample types should the test definitely work on and on which sample types would it be a bonus? Why?
  - What are the requirements for the test to meet this?
  - If liquid biopsies mentioned:
    - What type of liquid biopsy (saliva, blood, urine, csf)?
    - For which application(s)?
    - What advantages/disadvantages can liquid biopsies have?
    - In what areas is it possible for liquid biopsies to (partially) replace tissue biopsies? Where do tissue biopsies remain a must?

        - What does the implementation of liquid biopsy testing require from your laboratory?
- Do you think it is important for a molecular technique to be able to test multiple samples at the same time? Why?
- Do you think it is important for a molecular technique to be able to test multiple biomarkers at the same time? Why?
- What are the requirements for sample preparation?
    - How many steps is a maximum? How much time is a maximum?
- To what extent should a new test be automated? Does it need to be fully/semi-automated?
- What is desirable; the machine that does the interpretation of the data , machine interpretation with supervision of the analysis (requires explainability of the machine), or obtaining raw data and interpreting it yourself?
- Which device size is ideal/acceptable? Do you prefer a portable device or tabletop model?
- Which equipment features do you prefer? (e.g. buttons vs touchscreen, type of display, connected to the internet...)
- What do you think is more important within the various applications: sensitivity (TP/TP+FN) (absence of false negatives) or specificity (TN/TN+FP) (absence of false positives)?
    - What is the minimum diagnostic sensitivity and specificity (in general or per application)?
- Do you regret that your practical work is gradually being replaced by devices?
- How do you see the current clinical practice in oncology in general and in the field of molecular diagnostics evolving? What will it be like in, say, 10 years?
- Is there anything else you would like to add?

Industry (diagnostic/CRO)

1. General information

- Which organization and department/team do you work for?
- Could you briefly describe your job and your main tasks?
- How many years of experience do you currently have?
- What did you graduate in?

2. Current situation

- What is the current role of molecular diagnostics in clinical oncology?
- Which molecular diagnostic techniques/tests are you involved in or interested in? How/why?
    - What are their pros/cons?
    - For which applications are these tests used?
    - For which patient population are these important?
    - To what extent are these tests automated?
    - How do these tests give a result? Are these already fully interpreted results, e.g. biomarker present/absent, does it give raw data or a combination?
    - How many samples are (or can be) tested at the same time with these techniques?
    - How many biomarkers are/can be tested at the same time with these techniques?
    - To which sample types and subtypes do these tests apply? E.g. tissue: FFPE, FFT, etc.; blood: PMBCs, plasma, serum, ctDNA, CTC, etc.
        - If liquid biopsies apply:
            - For which application(s) is molecular testing done with this test on a liquid biopsy (primary diagnosis, monitoring of therapy response, minimal residual disease testing, analysis of therapy resistance)?
            - In your opinion, are these tests clinically validated (level 1) or are they still in a clinical research phase?
- Do you know what other molecular tests are relevant for cancer patients?
    - What are the pros and cons of these tests?
- Do you have an idea of the current average turnaround time and/or hands-on time of molecular tests?
    - Are you satisfied with this?
    - What is acceptable as hands-on time/TAT?
    - What is ideal and achievable as hands-on time/TAT?
- Do you believe that molecular diagnostic tests should be centralized in a network?
    - What do you think could be an advantage of partial or fully decentralized testing?
    - What are the requirements for a test that it could still be decentralized?

3. Demand

- What is important for a newly developed molecular test to implement in practice?
- What regulations does a new test need to meet in order to implement it? (ISO, IVDR, CE)
    - How are they obtained?
- In your opinion, within your field of interest, where do you currently see the most need for a new molecular test?
    - For which application(s) and/or on which (type(s) of) biomarker(s)?
    - What are the requirements for the technology to meet this need?
        - E.g. in relation to speed, cost, sensitivity/specificity, sample type, qualitative/semi-quantitative/absolute quantitative

- Do you think there is a need for a point-of-care test for complex molecular diagnostics? Why (not)? What are pros and cons?
    - What are the requirements for the technique here?
- Which sample types should the test definitely work on and on which sample types would it be a bonus? Why?
    - What are the requirements for the test to meet this?
    - Liquid biopsies mentioned:
        - What type of liquid biopsy (saliva, blood, urine, csf)?
        - For which application(s)?
        - What advantages/disadvantages can liquid biopsies have?
        - In what areas is it possible for liquid biopsies to (partially) replace tissue biopsies? Where do tissue biopsies remain a must?
- Do you think it is important for a molecular technique to be able to test multiple samples at the same time? Why?
- Do you think it is important for a molecular technique to be able to test multiple biomarkers at the same time? Why?
- What is the maximum duration of a training course to be able to perform a new test?
- To what extent should a new test be automated? Does it need to be fully/semi-automated?
- What is desirable; the machine that does the interpretation of the data, machine interpretation with supervision of the analysis (requires explainability of the machine), or obtaining raw data and interpreting it yourself?
- Which device size is ideal/acceptable? Do you prefer a portable or tabletop model?
- Which equipment features do you prefer? (e.g. buttons vs touchscreen, type of display, connected to the internet...)
- What do you think is more important within the various applications: sensitivity (TP/TP+FN) (absence of false negatives) or specificity (TN/TN+FP) (absence of false positives)?
    - What is the minimum diagnostic sensitivity and specificity?
- How do you see the current clinical practice in oncology in general and in the field of molecular diagnostics evolving? What will it be like in, say, 10 years?
- Is there anything else you would like to add?

Health policy and quality assessments organisations

1. General information

- Which organization and department/team do you work for?
- Could you briefly describe your job and your main tasks?
- How many years of experience do you currently have?
- What did you graduate in?

2. Current situation

- What is the current role of molecular diagnostics in clinical oncology?
- Which molecular techniques are currently used in clinical oncology?
    - What are their pros and cons?
    - For which applications are these techniques used?
    - For which patient population are these important?
- On which sample types and subtypes are molecular tests performed? (e.g. tissue: FFPE, FFT, etc.; blood: PMBCs, plasma, serum, ctDNA, CTC, etc.)
    - If liquid biopsies are used:
        - For which application(s) is molecular testing done on a liquid biopsy (primary diagnosis, monitoring of therapy response, minimal residual disease testing, analysis of therapy resistance)?
        - How often is this done?
        - In your opinion, are these tests clinically validated (level 1) or are they still in a clinical research phase?
    - Liquid biopsies are not yet used in routine clinical diagnostics, what do you think is needed to achieve this? (e.g. clinical validation, reimbursement)
- Do you have an idea of the current average turnaround time and/or hands-on time of molecular tests?
    - Are you satisfied with this?
    - What is acceptable as hands-on time/TAT?
    - What is ideal and achievable as hands-on time/TAT?
- Do you believe that molecular diagnostic tests should be centralized in a network?
    - How is this for the currently used tests?
    - What do you think could be an advantage of partial or fully decentralized testing?
    - What are the requirements for a test that it could still be decentralized?

3. Demand

- What is important for a newly developed molecular test to implement in practice?
- What regulations does a new test need to meet in order for it to be implemented in practice? (ISO, IVDR, CE)
    - How are they obtained?
- In your opinion, where do you currently see the most need for a new molecular test?
    - For which application(s) and/or on which (type(s) of) biomarker(s)?
    - What are the requirements for the technology to meet this need?
        - E.g. in relation to speed, cost, sensitivity/specificity, sample type, qualitative/semi-quantitative (low-medium-high)/absolute quantitative
- Do you think there is a need for a point-of-care test for complex molecular diagnostics? Why (not)? What are pros and cons?
    - What are the requirements for the technique here? E.g. about the complexity of the result; Should the result be unambiguous or should there be room for interpretation?

- Which sample types should the test definitely work on and on which sample types would it be a bonus? Why?
    - What are the requirements for the test to meet this?
    - If liquid biopsies mentioned:
        - What type of liquid biopsy (saliva, blood, urine, csf)?
        - For which application(s)?
        - What advantages/disadvantages can liquid biopsies have?
        - In what areas is it possible for liquid biopsies to (partially) replace tissue biopsies? Where do tissue biopsies remain a must?
- Do you think it is important for a molecular technique to be able to test multiple samples at the same time? Why?
- Do you think it is important for a molecular technique to be able to test multiple biomarkers at the same time? Why?
- To what extent should a new test be automated? Does it need to be fully/semi-automated?
- What is desirable; the machine that does the interpretation of the data, machine interpretation with supervision of the analysis (requires explainability of the machine), or obtaining raw data and interpreting it yourself?
- Which device size is ideal/acceptable? Do you prefer a portable device or tabletop model?
- What do you think is more important within the various applications: sensitivity (TP/TP+FN) (absence of false negatives) or specificity (TN/TN+FP) (absence of false positives)?
    - What is the minimum diagnostic sensitivity and specificity (in general or per application)?
- How do you see the current clinical practice in oncology in general and in the field of molecular diagnostics evolving? What will it be like in, say, 10 years?
- Is there anything else you would like to add?
