# Supplementary material for: Bridging the gap: Multi‐stakeholder perspectives of molecular diagnostics in oncology
Source: Mol Oncol. 2025 Aug 14;20(2):464–79. doi: 10.1002/1878-0261.70103 (PMC12936412; doi:10.1002/1878-0261.70103)
Supplement: Supplementary file 2 — Appendix S2. Online survey. [file MOL2-20-464-s001.pdf]

Universiteit Antwerpen

Bridging the Gap: A Stakeholder Analysis of Molecular Diagnostics in Oncology

Informed Consent

Dear participant,

You wish to participate in a scientific study on the unmet needs within molecular diagnostics in oncology and the requirements for new molecular technologies, carried out by Jorine Arnouts under the direction of Prof. Marc Peeters, Prof. Senada Koljenovic, Prof. Karolien De Wael, Prof. Karen Zwaenepoel, Prof. Timon Vandamme, and Dr. Greetje Vanhoutte. In a moment, we will present you a questionnaire. This will take about 10 minutes. **This is an anonymous study: you are never recognizable** during the course of this study, nor in the data we collect. If necessary, all data from this study will be digitized and stored for 10 years after the end of the study. **You have the right to refuse to participate and to stop your participation at any time.** You do not have to explain this and this will not entail any disadvantage or loss of benefits. All data already collected at that time can be used for data analysis. **The researchers of this study have the right to share anonymous databases** (in which participants are not recognizable to anyone) **with (inter)national colleagues** in the context of (this and/or later) scientific research. These data files are not accessible to other parties, including yourself or companies/organizations that do not fit within scientific research institutions. If you have any further questions or comments, please contact Jorine.Arnouts@uantwerpen.be

Best regards,
Jorine Arnouts

I have read and understood the information in the information document and (tick as appropriate):

- ○ I voluntarily agree to participate.
- ○ I do not wish to participate in this study.

Universiteit Antwerpen

Q1 What is your main speciality?

- ◯ Oncologist
- ◯ Pathologist
- ◯ Molecular biologist
- ◯ Clinical biologist
- ◯ Laboratory technician
- ◯ Health quality organization
- ◯ Health policy organization
- ◯ Industry
- ◯ Other(s), please specify: ……………………………………………….

Q1.1 What is your subspeciality? Multiple answers possible.

- ☐ Brain
- ☐ Breast
- ☐ Cholangiocarcinoma
- ☐ Colorectal
- ☐ Endometrium
- ☐ Esophageal and gastric
- ☐ GIST
- ☐ Haemato
- ☐ Head and neck
- ☐ Lung
- ☐ Melanoma
- ☐ Ovary
- ☐ Pancreas
- ☐ Prostate
- ☐ Sarcomas
- ☐ Thryoid
- ☐ Urothelial carcinoma
- ☐ Other(s), please specify: ………………………………………………….

Universiteit Antwerpen

☐ Not applicable

Display this question:

If What is your main speciality? = Oncologist

Or What is your main speciality? = Pathologist

Or What is your main speciality? = Molecular biologist

Or What is your main speciality? = Clinical biologist

Or What is your main speciality? = Laboratory technician

Q1.2 In what type of center do you currently work?

○ University hospital

○ Non-university hospital

○ (Private) laboratory not present within a hospital's infrastructure

○ Other(s), please specify: ……………………………………………………

Display this question:

If What is your main speciality? = Industry

Q1.3 In which branch of oncology do you work?

○ Diagnostic

○ Therapeutic

○ Contract research organization (CRO)

○ Other(s), please specify: ……………………………………………………

Q2 Is molecular testing performed in your center?

○ Yes

○ No

Universiteit Antwerpen

Deze vraag weergeven:

If Is molecular testing performed in your center? = Yes

Q2.1 Which molecular techniques are available in your center?

- [ ] Whole Genome Sequencing (WGS)
- [ ] Whole Exome Sequencing (WES)
- [ ] Targeted Next-Generation Sequencing (NGS)
- [ ] Quantitative PCR
- [ ] Droplet digital PCR
- [ ] Fast PCR (e.g. Idylla)
- [ ] Fluorescence in-situ hybridization (FISH)
- [ ] Microarray
- [ ] Others, please specify: ……………………………………………………………

Q3 Which country do you currently work in?

▼ Afghanistan (1) ... Zimbabwe (1357)

Universiteit Antwerpen

Q4 How many years of experience do you have (If applicable: including internships during specialization)?

- ◯ ≤ 5 years
- ◯ 6-10 years
- ◯ 11-15 years
- ◯ 16-20 years
- ◯ > 20 years

For your information:
Diagnostic biomarker: Used to detect or confirm the presence of cancer or identify its subtype.
Prognostic biomarker: Used to give information on the patient's overall cancer outcomes with or without standard treatment.
Predictive biomarker: Used to understand which treatment the patient is most likely to respond to or benefit from.
Therapy respone, follow-up or monitoring biomarker: Used to monitor cancer patients over time for treatment response, disease progression, or recurrence.
Screening biomarker: Used to detect the presence of cancer or identify individuals at risk of developing cancer in asymptomatic (high-risk) populations.
Minimal Residual Disease (MRD) biomarker: Used to detect and quantify residual cancer cells after treatment, aiding in outcome prediction and treatment monitoring. Also called Measurable Residual Disease or Molecular Residual Disease.

Universiteit Antwerpen

Q5 Please select your level of agreement with the following statements: *The **most important unmet need** within current clinical molecular diagnostics in oncology is testing ...*

	Fully disagree	Somewhat disagree	Neutral	Somewhat agree	Fully agree
Diagnostic biomarkers on liquid biopsy samples	○	○	○	○	○
Prognostic biomarkers on liquid biopsy samples	○	○	○	○	○
Predictive biomarkers on liquid biopsy samples	○	○	○	○	○
Therapy response biomarkers	○	○	○	○	○
Screening biomarkers	○	○	○	○	○
Minimal Residual Disease (MRD) biomarkers	○	○	○	○	○

Universiteit Antwerpen

Q6 Please select your level of agreement with the following statements: *Testing ... will be* ***implemented in the clinic*** *within the* ***next 5-10 years****.*

	Fully disagree	Somewhat disagree	Neutral	Somewhat agree	Fully agree
Diagnostic biomarkers on liquid biopsy samples	○	○	○	○	○
Prognostic biomarkers on liquid biopsy samples	○	○	○	○	○
Predictive biomarkers on liquid biopsy samples	○	○	○	○	○
Therapy response biomarkers	○	○	○	○	○
Screening biomarkers	○	○	○	○	○
Minimal Residual Disease (MRD) biomarkers	○	○	○	○	○

Universiteit Antwerpen

Q7 Please select **turnaround time* and multiplexing**** preferences for the following applications.
**From request of molecular test to obtaining a report. **Simultaneous detection and analysis of multiple molecular targets within a single assay.*

	Single-biomarker, 1 day	Small biomarker panel (<10), 2-5 days	Big biomarker panel (e.g. targeted NGS), > 1 week	Comprehensive testing (e.g. WGS), > 2 weeks	Not sure
Diagnostic biomarkers	○	○	○	○	○
Prognostic biomarkers	○	○	○	○	○
Predictive biomarkers	○	○	○	○	○
Therapy response biomarkers	○	○	○	○	○
Screening biomarkers	○	○	○	○	○
Minimal Residual Disease (MRD) biomarkers	○	○	○	○	○
Molecular testing of samples from patients with acute illness in need for rapid treatment	○	○	○	○	○
If other(s) or any exception(s), please specify:	○	○	○	○	○

Q8 Please indicate the **importance** of a novel molecular technique, expected to enter the market in the next 5-10 years, **being able to test on** ((cell-free) nucleic acids of) **the following matrices**:

	Not important	Not very important	Neutral	Reasonably important	Very important
Fresh frozen tissue	○	○	○	○	○
Formalin-fixed paraffin-embedded (FFPE) tissue	○	○	○	○	○
Cytology samples (e.g. fine-needle aspirates, fluid aspirations, smears etc.)	○	○	○	○	○
Plasma	○	○	○	○	○
Serum	○	○	○	○	○
Urine	○	○	○	○	○
Cerebrospinal fluid (csf)	○	○	○	○	○
Saliva	○	○	○	○	○
Faeces	○	○	○	○	○
Other(s), please specify:	○	○	○	○	○

Q9 Should all novel molecular techniques be **centralized**?

◯ Yes

◯ No

◯ Not sure

Display this question:

If Should all novel molecular techniques be centralized? = No

Q9.1 Drag the **top 3 must-have characteristics** of **molecular techniques employed in decentralized setting** in the box and rank them (1 = most, 3 = least important).

Items	Top 3 Must-have
Short turnaround time	
Short hands-on time	
Low costs	
Easy hands-on work	
Easy data-analysis	
Comprehensive multiplexing	
High throughput	

Display this question:

If Should all novel molecular techniques be centralized? = No

Q9.2 Drag the **top 3 must-have characteristics** of **centralized molecular techniques** in the box and rank them (1 = most, 3 = least important).

Items	Top 3 Must-have
Short turnaround time	
Short hands-on time	
Low costs	
Easy hands-on work	
Easy data-analysis	
Comprehensive multiplexing	
High throughput	

Q10 Drag the **top 3 must-have characteristics** of molecular techniques for detecting **diagnostic biomarkers** in the box and rank them (1 = most, 3 = least important).

Items	Top 3 Must-have
Short turnaround time	
Low costs	
Easy hands-on work	
Easy data analysis	
Comprehensive multiplexing	
Quantitative results	
High throughput	
Possible on liquid biopsies	

Q11 Drag the **top 3 must-have characteristics** of molecular techniques for detecting **prognostic biomarkers** in the box and rank them (1 = most, 3 = least important).

Items	Top 3 Must-have
Short turnaround time	
Low costs	
Easy hands-on work	
Easy data analysis	
Comprehensive multiplexing	
Quantitative results	
High throughput	
Possible on liquid biopsies	

Q12 Drag the **top 3 must-have characteristics** of molecular techniques for detecting **predictive biomarkers** in the box and rank them (1 = most, 3 = least important).

Items	Top 3 Must-have
Short turnaround time	
Low costs	
Easy hands-on work	
Easy data analysis	
Comprehensive multiplexing	
Quantitative results	
High throughput	
Possible on liquid biopsies	

Universiteit Antwerpen

Q13 Drag the **top 3 must-have characteristics** of molecular techniques for detecting **therapy response biomarkers** in the box and rank them (1 = most, 3 = least important).

Items	Top 3 Must-have
Short turnaround time	
Low costs	
Easy hands-on work	
Easy data analysis	
Comprehensive multiplexing	
Quantitative results	
High throughput	
Possible on liquid biopsies	

Q14 Drag the **top 3 must-have characteristics** of molecular techniques for detecting **screening biomarkers** into the box and rank them by importance (1 = most, 3 = least important).

Items	Top 3 Must-have
Short turnaround time	
Low costs	
Easy hands-on work	
Easy data analysis	
Comprehensive multiplexing	
Quantitative results	
High throughput	
Possible on liquid biopsies	

Universiteit Antwerpen

Q15 Drag the **top 3 must-have characteristics** of molecular techniques for detecting **Minimal Residual Disease (MRD) biomarkers** in the box and rank them (1 = most, 3 = least important).

Items	Top 3 Must-have
Short turnaround time	
Low costs	
Easy hands-on work	
Easy data analysis	
Comprehensive multiplexing	
Quantitative results	
High throughput	
Possible on liquid biopsies	

Q16 Drag the **top 3 must-have characteristics** of molecular techniques for testing **samples from patients with acute illness in need for rapid treatment** in the box and rank them (1 = most, 3 = least important).

Items	Top 3 Must-have
Short turnaround time	
Low costs	
Easy hands-on work	
Easy data analysis	
Comprehensive multiplexing	
Quantitative results	
High throughput	
Possible on liquid biopsies	

Is there anything you would like to add?

……
……
……
…………………………………………………………………………………….

By clicking the next arrow, you consent to submit this survey.
