# Supplementary material for: Bridging the gap: Multi‐stakeholder perspectives of molecular diagnostics in oncology
Source: Mol Oncol. 2025 Aug 14;20(2):464–79. doi: 10.1002/1878-0261.70103 (PMC12936412; doi:10.1002/1878-0261.70103)
Supplement: Supplementary file 3 — Table S1. Survey results on emerging applications as unmet needs in molecular oncology and their likelihood for clinical implementation. [file MOL2-20-464-s002.docx]

Table S1. Survey results on emerging applications as unmet needs in molecular oncology and their likelihood for clinical implementation

| **Testing diagnostic biomarkers on LB** | | | | | | | | | | |
| --- | --- | --- | --- | --- | --- | --- | --- | --- | --- | --- |
|  | *Most important unmet need* | | | | | *Likelihood near-future implementation* | | | | |
|  | n | % | Mean score | SD | Rank | n | % | Mean score | SD | Rank |
|  |  |  | 4.05 | 1.041 | **3** |  |  | 4.12 | 1.008 | **3** |
| *Fully disagree* | 1 | 0.9 |  |  |  | 1 | 0.9 |  |  |  |
| *Somewhat disagree* | 11 | 9.5 |  |  |  | 9 | 7.8 |  |  |  |
| *Neutral* | 15 | 12.9 |  |  |  | 14 | 12.1 |  |  |  |
| *Somewhat agree* | 33 | 28.4 |  |  |  | 33 | 28.4 |  |  |  |
| *Fully agree* | 45 | 38.8 |  |  |  | 47 | 40.5 |  |  |  |
| *Missing* | 11 | 9.5 |  |  |  | 12 | 10.3 |  |  |  |
| **Testing prognostic biomarkers on LB** | | | | | | | | | | |
|  | *Most important unmet need* | | | | | *Likelihood near-future implementation* | | | | |
|  | n | % | Mean score | SD | Rank | n | % | Mean score | SD | Rank |
|  |  |  | 3.68 | 1.036 | **5** |  |  | 4.04 | 0.843 | **5** |
| *Fully disagree* | 3 | 2.6 |  |  |  | 0 | 0 |  |  |  |
| *Somewhat disagree* | 11 | 9.5 |  |  |  | 6 | 5.2 |  |  |  |
| *Neutral* | 26 | 22.4 |  |  |  | 17 | 14.7 |  |  |  |
| *Somewhat agree* | 40 | 34.5 |  |  |  | 49 | 42.2 |  |  |  |
| *Fully agree* | 24 | 20.7 |  |  |  | 33 | 28.4 |  |  |  |
| *Missing* | 12 | 10.3 |  |  |  | 11 | 9.5 |  |  |  |
| **Testing predictive biomarkers on LB** | | | | | | | | | | |
|  | *Most important unmet need* | | | | | *Likelihood near-future implementation* | | | | |
|  | n | % | Mean score | SD | Rank | n | % | Mean score | SD | Rank |
|  |  |  | 4.15 | 0.984 | **1** |  |  | 4.14 | 0.897 | **2** |
| *Fully disagree* | 2 | 1.7 |  |  |  | 0 | 0 |  |  |  |
| *Somewhat disagree* | 4 | 3.4 |  |  |  | 5 | 4.3 |  |  |  |
| *Neutral* | 19 | 16.4 |  |  |  | 20 | 17.2 |  |  |  |
| *Somewhat agree* | 30 | 25.9 |  |  |  | 34 | 29.3 |  |  |  |
| *Fully agree* | 48 | 41.4 |  |  |  | 45 | 38.8 |  |  |  |
| *Missing* | 13 | 11.2 |  |  |  | 12 | 10.3 |  |  |  |
| **Testing therapy response biomarkers** | | | | | | | | | | |
|  | *Most important unmet need* | | | | | *Likelihood near-future implementation* | | | | |
|  | n | % | Mean score | SD | Rank | n | % | Mean score | SD | Rank |
|  |  |  | 4.13 | 0.904 | **2** |  |  | 4.25 | 0.809 | **1** |
| *Fully disagree* | 1 | 0.9 |  |  |  | 0 | 0 |  |  |  |
| *Somewhat disagree* | 5 | 4.3 |  |  |  | 2 | 1.7 |  |  |  |
| *Neutral* | 15 | 12.9 |  |  |  | 17 | 14.7 |  |  |  |
| *Somewhat agree* | 41 | 35.3 |  |  |  | 35 | 30.2 |  |  |  |
| *Fully agree* | 41 | 35.3 |  |  |  | 46 | 39.7 |  |  |  |
| *Missing* | 13 | 11.2 |  |  |  | 16 | 13.8 |  |  |  |
| **Testing MRD biomarkers** | | | | | | | | | | |
|  | *Most important unmet need* | | | | | *Likelihood near-future implementation* | | | | |
|  | n | % | Mean score | SD | Rank | n | % | Mean score | SD | Rank |
|  |  |  | 4.15 | 0.944 | **1** |  |  | 4.06 | 0.906 | **4** |
| *Fully disagree* | 2 | 1.7 |  |  |  | 0 | 0 |  |  |  |
| *Somewhat disagree* | 2 | 1.7 |  |  |  | 7 | 6 |  |  |  |
| *Neutral* | 21 | 18.1 |  |  |  | 18 | 15.5 |  |  |  |
| *Somewhat agree* | 32 | 27.6 |  |  |  | 40 | 34.5 |  |  |  |
| *Fully agree* | 46 | 39.7 |  |  |  | 38 | 32.8 |  |  |  |
| *Missing* | 13 | 11.2 |  |  |  | 13 | 11.2 |  |  |  |
| **Testing screening biomarkers** | | | | | | | | | | |
|  | *Most important unmet need* | | | | | *Likelihood near-future implementation* | | | | |
|  | n | % | Mean score | SD | Rank | n | % | Mean score | SD | Rank |
|  |  |  | 3.97 | 1.11 | **4** |  |  | 3.75 | 1.13 | **6** |
| *Fully disagree* | 5 | 4.3 |  |  |  | 3 | 2.6 |  |  |  |
| *Somewhat disagree* | 6 | 5.2 |  |  |  | 15 | 12.9 |  |  |  |
| *Neutral* | 17 | 14.7 |  |  |  | 19 | 16.4 |  |  |  |
| *Somewhat agree* | 35 | 30.2 |  |  |  | 35 | 30.2 |  |  |  |
| *Fully agree* | 41 | 35.3 |  |  |  | 32 | 27.6 |  |  |  |
| *Missing* | 12 | 10.3 |  |  |  | 12 | 10.3 |  |  |  |
