# Supplementary material for: Bridging the gap: Multi‐stakeholder perspectives of molecular diagnostics in oncology
Source: Mol Oncol. 2025 Aug 14;20(2):464–79. doi: 10.1002/1878-0261.70103 (PMC12936412; doi:10.1002/1878-0261.70103)
Supplement: Supplementary file 4 — Table S2. Preferred characteristics for technologies in decentralized and centralized settings. [file MOL2-20-464-s003.docx]

Table S2. Preferred characteristics for technologies across different applications

|  | Mean ranking score | SD | Rank |
| --- | --- | --- | --- |
| **Diagnostic biomarkers** |  |  |  |
| *Short TAT* | 2.67 | 1.36 | **1** |
| *Low costs* | 3.54 | 0.84 | **4** |
| *Easy hands-on work* | 3.80 | 0.56 | **7** |
| *Easy data-analysis* | 3.65 | 0.71 | **6** |
| *Comprehensive multiplexing* | 3.50 | 0.97 | **3** |
| *Quantitative results* | 3.65 | 0.82 | **6** |
| *High throughput* | 3.56 | 0.93 | **5** |
| *On liquid biopsies* | 3.19 | 1.11 | **2** |
| **Prognostic biomarkers** |  |  |  |
| *Short TAT* | 3.38 | 1.01 | **7** |
| *Low costs* | 2.09 | 0.63 | **4** |
| *Easy hands-on work* | 2.25 | 0.87 | **6** |
| *Easy data-analysis* | 2.03 | 0.81 | **3** |
| *Comprehensive multiplexing* | 1.72 | 0.85 | **1** |
| *Quantitative results* | 1.87 | 0.76 | **2** |
| *High throughput* | 2.24 | 0.83 | **5** |
| *On liquid biopsies* | 3.37 | 1.09 | **6** |
| **Predictive biomarkers** |  |  |  |
| *Short TAT* | 3.01 | 1.30 | **1** |
| *Low costs* | 3.55 | 0.81 | **5** |
| *Easy hands-on work* | 3.70 | 0.83 | **7** |
| *Easy data-analysis* | 3.55 | 0.79 | **5** |
| *Comprehensive multiplexing* | 3.54 | 0.93 | **4** |
| *Quantitative results* | 3.41 | 1.09 | **3** |
| *High throughput* | 3.64 | 0.85 | **6** |
| *On liquid biopsies* | 3.28 | 1.02 | **2** |
| **Therapy response biomarkers** |  |  |  |
| *Short TAT* | 3.10 | 1.20 | **2** |
| *Low costs* | 3.43 | 0.97 | **4** |
| *Easy hands-on work* | 3.83 | 0.55 | **8** |
| *Easy data-analysis* | 3.51 | 0.81 | **5** |
| *Comprehensive multiplexing* | 3.65 | 0.84 | **6** |
| *Quantitative results* | 3.28 | 1.14 | **3** |
| *High throughput* | 3.77 | 0.75 | **7** |
| *On liquid biopsies* | 3.04 | 1.17 | **1** |
| **Screening biomarkers** |  |  |  |
| *Short TAT* | 3.52 | 1.03 | **4** |
| *Low costs* | 2.80 | 1.21 | **1** |
| *Easy hands-on work* | 3.75 | 0.67 | **6** |
| *Easy data-analysis* | 3.68 | 0.72 | **5** |
| *Comprehensive multiplexing* | 3.75 | 0.75 | **6** |
| *Quantitative results* | 3.68 | 0.77 | **5** |
| *High throughput* | 3.37 | 1.08 | **3** |
| *On liquid biopsies* | 3.08 | 1.11 | **2** |
| **MRD biomarkers** |  |  |  |
| *Short TAT* | 3.35 | 1.12 | **3** |
| *Low costs* | 3.55 | 0.88 | **4** |
| *Easy hands-on work* | 3.84 | 0.54 | **7** |
| *Easy data-analysis* | 3.55 | 0.75 | **4** |
| *Comprehensive multiplexing* | 3.66 | 0.85 | **5** |
| *Quantitative results* | 2.99 | 1.25 | **1** |
| *High throughput* | 3.67 | 0.81 | **6** |
| *On liquid biopsies* | 3.11 | 1.16 | **2** |
| **Acute illness in need for rapid treatment** | |  |  |
| *Short TAT* | 2.19 | 1.36 | **1** |
| *Low costs* | 3.77 | 0.68 | **6** |
| *Easy hands-on work* | 3.54 | 0.88 | **4** |
| *Easy data-analysis* | 3.51 | 0.75 | **3** |
| *Comprehensive multiplexing* | 3.71 | 0.71 | **5** |
| *Quantitative results* | 3.80 | 0.65 | **7** |
| *High throughput* | 3.77 | 0.66 | **6** |
| *On liquid biopsies* | 3.32 | 0.91 | **2** |
