# Supplementary material for: Bridging the gap: Multi‐stakeholder perspectives of molecular diagnostics in oncology
Source: Mol Oncol. 2025 Aug 14;20(2):464–79. doi: 10.1002/1878-0261.70103 (PMC12936412; doi:10.1002/1878-0261.70103)
Supplement: Supplementary file 5 — Table S3. Preferred characteristics for technologies across different applications. [file MOL2-20-464-s005.docx]

Table S3. Preferred characteristics for technologies in decentralized and centralized settings

|  | Mean ranking score | SD | Rank |
| --- | --- | --- | --- |
| **Decentralized technologies** |  |  |  |
| *Short TAT* | 1.9 | 1.25 | **1** |
| *short hands-on time* | 3.69 | 0.69 | **6** |
| *Low costs* | 3.28 | 0.94 | **4** |
| *Easy hands-on work* | 4.41 | 0.99 | **7** |
| *Easy data-analysis* | 3.03 | 0.96 | **2** |
| *Comprehensive multiplexing* | 3.26 | 1.09 | **3** |
| *High throughput* | 3.44 | 0.97 | **5** |
| **Centralized technologies** |  |  |  |
| *Short TAT* | 2.74 | 1.25 | **3** |
| *short hands-on time* | 3.79 | 0.66 | **6** |
| *Low costs* | 3.05 | 1.03 | **4** |
| *Easy hands-on work* | 3.85 | 0.49 | **7** |
| *Easy data-analysis* | 3.67 | 0.81 | **5** |
| *Comprehensive multiplexing* | 2.44 | 1.23 | **1** |
| *High throughput* | 2.46 | 1.12 | **2** |
